# Microwave-Assisted Synthesis of Fluorescent Pyrido[2,3-*b*]indolizines from Alkylpyridinium Salts and Enaminones

**DOI:** 10.3390/molecules25184059

**Published:** 2020-09-05

**Authors:** Ekaterina A. Sokolova, Alexey A. Festa, Karthikeyan Subramani, Victor B. Rybakov, Alexey V. Varlamov, Leonid G. Voskressensky, Erik V. Van der Eycken

**Affiliations:** 1 Organic Chemistry Department, Science Faculty, RUDN University, Miklukho-Maklaya st., 6, 117198 Moscow, Russia; soko-katya@mail.ru (E.A.S.); festa_aa@pfur.ru (A.A.F.); subramanikarthikeya91@gmail.com (K.S.); avarlamov@sci.pfu.edu.ru (A.V.V.); lvoskressensky@sci.pfu.edu.ru (L.G.V.); 2 Department of Chemistry, Lomonosov Moscow State University, Leninskie Gory, 1–3, 119991 Moscow, Russia; rybakov20021@yandex.ru; 3 Laboratory for Organic & Microwave-Assisted Chemistry (LOMAC), Department of Chemistry, University of Leuven (KU Leuven), Celestijnenlaan 200F, B-3001 Leuven, Belgium

**Keywords:** pyridinium ylide, indolizine, domino reaction, fluorescence

## Abstract

Pyridinium ylides are well recognized as dipoles for cycloaddition reactions. In its turn, the microwave-assisted interaction of *N*-(cyanomethyl)-2-alkylpyridinium salts with enaminones unexpectedly proceeds as a domino sequence of cycloisomerization and cyclocondensation reactions, instead of a 1,3-dipolar cycloaddition. The reaction takes place in the presence of sodium acetate as base and employs benign solvents. The optical properties of the resulting pyrido[2,3-*b*]indolizines were studied, showing green light emission with high fluorescence quantum yields.

## 1. Introduction

Indolizines, and in particular the annulated ones, are frequently found to exhibit useful biological [1,2,3,4,5,6] and optical properties [7,8,9,10,11,12,13,14]. The synthesis of indolizines usually relies on the reactivity of pyridinium ylides, which can undergo cycloaddition reactions with alkenes [15,16,17,18] or alkynes [19,20,21,22,23]. In some cases, interaction of ylides with alkenes or alkynes leads to a different outcome [24,25,26]. Another approach towards the indolizine scaffold is based on intramolecular cyclization of 2-alkylpyridinium ylides. For instance, Opatz et al. used 2-alkyl-1-(cyanomethyl)pyridinium salts to prepare aminoindolizines [27]. Following our interest in the chemistry of cyanomethylpyridinium salts [28,29,30], we recently showed that the interaction of *N*-(cyanomethyl)pyridinium chlorides with enaminones under basic conditions proceeds as a pseudo-three-component reaction, resulting in the formation of pyridoindolizine-10-carbonitriles [31].

**Scheme 1:**
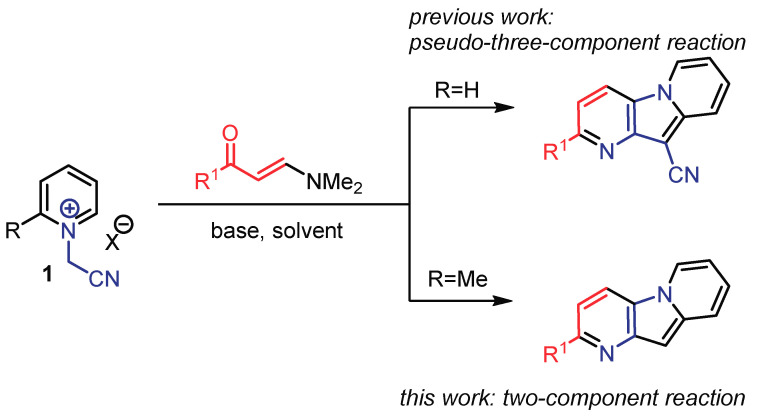
General representation of the current work.

In this work we discovered that the reactions of 2-alkyl-*N*-(cyanomethyl)pyridinium salts **1** with enaminones **2** proceed unexpectedly as a two-component domino sequence of cycloisomerization and cyclocondensation reactions, while cycloaddition processes were not observed (Scheme 1).

## 2. Results

Based on the previously investigated reaction [31], we started optimization of the conditions with the use of sodium acetate as base and an iso-propanol/water mixture as solvent (Table 1, entries 1–6). Varying the ratio of the starting materials and the base, the target compound **3a** was obtained with 50% yield (Table 1, entry 5). The use of other bases such as Et_3_N, DIPEA, or NH_4_OAc did not improve the yield (Table 1, entries 7–9). Inorganic bases did not ameliorate the process either (Table 1, entries 10 and 11). The variation of reaction time or temperature commonly led to diminished yields (Table 1, entries 12–14).

**Scheme 2:**
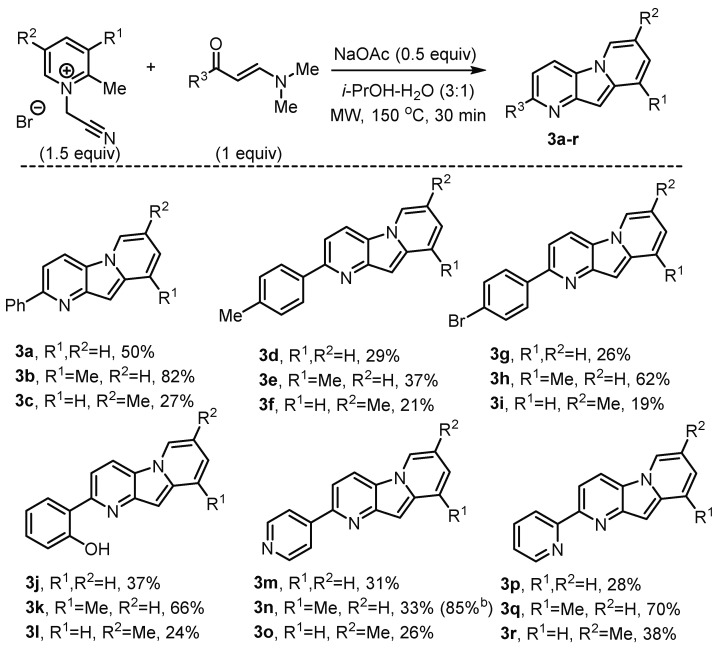
The scope of the reaction between *N*-cyanomethyl-2-methylpyridinium bromides and various enaminones ^a^. ^a^ General conditions: a mixture of pyridinium salt **1** (0.591 mmol), enaminone **2** (0.394 mmol), and sodium acetate (0.197 mmol) in isopropyl alcohol (3 mL) and water (1 mL) was placed into the microwave reactor and irradiated at 150 °C for 30 min. ^b^ The reaction was performed on 1.182 mmol scale of *N*-cyanomethyl-2,3-dimethylpyridinium salt.

With the optimized conditions in hand, we went on to investigate the reaction scope. It turned out that the reaction of *N*-cyanomethyl-2,3-dimethylpyridinium salt with enaminone was more effective, and the target product **3b** was isolated with 82% yield (Scheme 2). On the contrary, the interaction of 2,5-dimethylpyridinium salt with the enaminone delivered product **3c** with 27% yield. When *p*-methylphenyl-substituted enaminone was used, the compounds **3d–f** were isolated with 21–37% yield. Bromo-substituted enaminones could be also used with various pyridinium salts to give indolizines **3g–i** with 19–62% yield. Pyridoindolizines **3j–l** with a phenol moiety were prepared with 24–66% yield. Moreover, we were pleased to find the pyridyl-containing enaminones to work successfully, producing the corresponding compounds **3m–r** with poor to moderate yields. It is worth noting that taking *N*-cyanomethyl-2,3-dimethylpyridinium bromide in a large excess increased the yield of the compound **3n** from 33% to 85%. Unfortunately, increasing the loading of the pyridinium salts in other cases did not result in yield improvement. The use of aliphatic enaminones (R^3^ = Me or Et) in the reactions with *N*-cyanomethyl-2,3-dimethylpyridinium bromide generated complex mixtures, and no target product could be isolated. As a rule, the use of 2,3-dimethylpyridiniums resulted in greater yields of the target pyridoindolizines **3**. The scope of the enaminones included various aryl groups, even phenols and heterocycles, while the use of aliphatic enaminones was found to be a limitation of the method.

**Figure 1:**
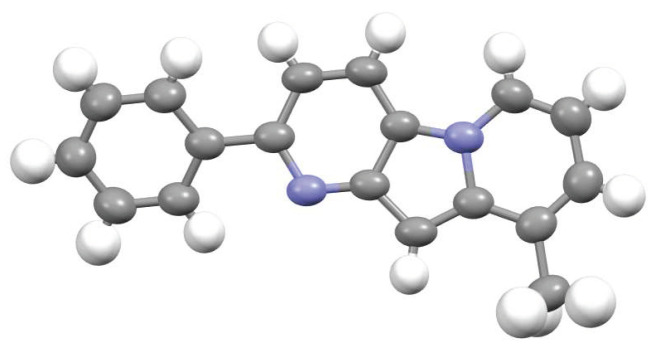
Crystal structure of **3b** (CCDC 1922817).

The structure of pyridoindolizine **3b** was unambiguously determined by a single crystal X-ray diffraction study (Figure 1, CCDC 1922817).

**Scheme 3:**
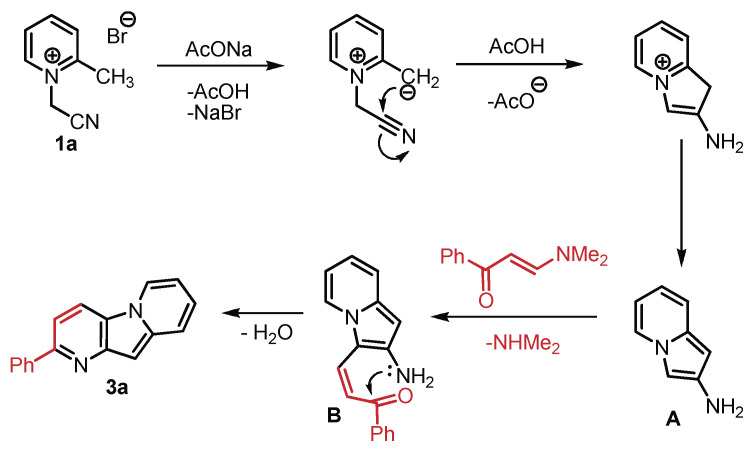
Proposed mechanism for pyrido[2,3-*b*]indolizine formation.

The reaction presumably starts with the intramolecular cyclization of a deprotonated α-methyl group on a nitrile moiety, eventually giving an aminoindolizine intermediate **A** [27] (Scheme 3). The interaction of the latter with enaminone produces an intermediate **B**. The cyclocondensation of **B** completes the reaction sequence, delivering pyridoindolizine **3a**. The intermediate **A** is evidently a highly nucleophilic species, containing a π-extensive pyrrole fragment combined with an amino group, readily reacting with the present electrophiles. Unfortunately, our attempts to isolate this intermediate failed. Even experiments in the absence of the enaminone generated multicomponent mixtures, pointing out the possibility for **A** to interact with the starting salt **1a**.

**Figure 2:**
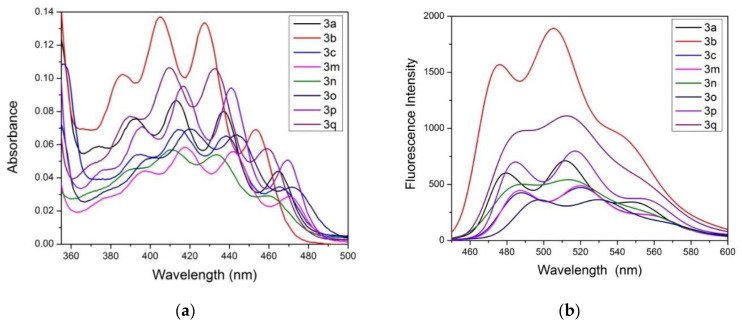
The absorbance (**a**) and emission (**b**) spectra of the above synthesized compounds in toluene.

The optical properties of the synthesized compounds were evaluated and all the spectra were measured in toluene solutions (Figure 2, Table 2, separate images are available in Supplementary Materials). Indolizines **3a–c**, **m–q** exhibited absorption peak maxima at 403–420 nm. The emission peak maxima lay in the green region 505–528 nm, and the largest Stokes shift 4950 cm^−1^ was registered for compound **3b**. The fluorescence quantum yields (FQYs) were determined using coumarin 153 as a standard [32]. The lowest FQY values of 55–63% were measured for 4-pyridyl-substituted pyridoindolizines **3m–o**, while the phenyl-substituted pyridoindolizine **3b** demonstrated the highest FQY, 82%. This optical behavior is in accordance with the literature. For instance, indolizines, condensed with isoindole [33], quinoline [34,35] or dihydropyrrole [36] cycles also emit in the blue to green region 410–556 nm with FQYs up to 77%.

## 3. Materials and Methods

### 3.1. General Information

Starting reagents were purchased from commercial sources and were used without any additional purification. Enaminones **2** were prepared according to the literature procedures [37]. Microwave reactions were conducted in a Monowave 300 Microwave Reactor (Anton Paar GmbH, Graz, Austria). Column chromatography was performed using silica gel (230–400 mesh) and mixtures in different proportions of ethyl acetate with hexane as the mobile phase. Melting points were determined on a SMP-10 apparatus (Barloworld Scientific Limited, Stone, UK). ^1^H NMR spectra were recorded on a 400 MHz spectrometer (Bruker, 100 MHz for ^13^C NMR) and referenced to the residual signals of the solvent (for ^1^H and ^13^C). Chemical shifts are reported in parts per million (δ/ppm). Coupling constants are reported in Hertz (*J*/Hz). The peak patterns are indicated as follows: s, singlet; d, doublet; t, triplet; q, quadruplet; m, multiplet; dd, doublet of doublets and td, triplet of doublets. Low resolution mass spectra were recorded with an LCMS-8040 triple quadrupole liquid chromatograph mass-spectrometer (Shimadzu corp., Tokyo, Japan). The reaction progress was monitored by TLC and the spots were visualized under UV light (254 or 365 nm). Elemental analysis was performed on a EuroVector EA-3000 instrument (EuroVector S.p.A., Milan, Italy).

### 3.2. General Procedure for the Synthesis of Salts **1a**–**c**

Bromoacetonitrile (0.026 mol) was added to a stirred solution of corresponding pyridine (0.022 mol) in acetonitrile (10 mL). The reaction mixture was heated at reflux for 4 h. The precipitate was filtered, washed with acetonitrile, and dried in vacuum over P_2_O_5_.

*N-(Cyanomethyl)-2-methylpyridinium bromide (***1a***)*. White powder; m.p. 195–196 °C (decomp.); yield, 3.56 g (76%); ^1^H NMR (400 MHz, CDCl_3_) δ 9.16 (1H, d, *J* = 6.1 Hz, H-6), 8.59–8.62 (1H, m, H-4), 8.16 (1H, d, *J* = 7.6 Hz, H-3), 8.06–8.08 (1H, m, H-5), 6.11 (2H, d, *J* = 1.5 Hz, CH_2_-CN), 2.90 (3H, s, C_2_-CH_3_). ^13^C NMR (100 MHz, CDCl_3_) δ 156.2, 147.3, 146.1, 130.2, 126.1, 113.5, 45.1, 20.0. ESI MS: *m/z* 133 [M]^+^. Elemental analysis calcd (%) for C_8_H_9_BrN_2_: C 45.10; H 4.26; N 13.15; found: C 45.02; H 4.29; N 13.26.

*N-(Cyanomethyl)-2,3-dimethylpyridinium bromide* (**1b**). Light beige powder; m.p. 175–177 °C (decomp.); yield, 3.60 g (72%); IR (cm^−1^): 2256 (CN); ^1^H NMR (400 MHz, CDCl_3_) δ 9.05 (1H, d, *J* = 6.1 Hz, H-6), 8.53 (1H, d, *J* = 8.1 Hz, H-4), 7.98–8.01 (1H, m, H-5), 6.20 (2H, s, CH_2_-CN), 2.83 (3H, s, C_2_-CH_3_), 2.52 (3H, s, C_3_-CH_3_). ^13^C NMR (100 MHz, CDCl_3_) δ 155.5, 147.3, 143.9, 139.2, 125.1, 113.7, 45.9, 19.3, 17.3. ESI MS: *m*/*z* 147 [M]^+^. Elemental analysis calcd (%) for C_9_H_11_BrN_2_: C 47.60; H 4.88; N 12.34; found: C 47.56; H 4.87; N 12.43.

*N-(Cyanomethyl)-2,5-dimethylpyridinium bromide* (**1c**). Light beige powder; m.p. 168–169 °C (decomp.); yield, 2.90 g (58%); ^1^H NMR (400 MHz, CDCl_3_) δ 9.11 (1H, s, H-6), 8.49 (1H, d, *J* = 8.3 Hz, H-3), 8.08 (1H, d, *J* = 8.3 Hz, H-4), 6.07 (2H, s, CH_2_-CN), 2.88 (3H, s, C_2_-CH_3_), 2.46 (3H, s, C_5_-CH_3_). ^13^C NMR (100 MHz, CDCl_3_) δ 153.2, 147.9, 145.3, 136.5, 129.5, 113.5, 44.9, 19.4, 17.4. ESI MS: *m*/*z* 147 [M]^+^. Elemental analysis calcd (%) for C_9_H_11_BrN_2_: C 47.60; H 4.88; N 12.34; found: C 47.57; H 4.91; N 12.45.

### 3.3. General Procedure for the Synthesis of Enaminones **2**

A mixture of dimethylformamide dimethylacetal (14.8 mmol) and methyl ketone (14.8 mmoL) was placed into the microwave reactor and irradiated at 150 °C for 15 min, then left to cool to room temperature. After cooling, the precipitate was filtered-off, washed twice with toluene and dried on air.

*(E)-3-(Dimethylamino)-1-phenylprop-2-en-1-one* (**2a**). Yellow powder; m.p. 91–92 °C; yield, 1.17 g (45%); ^1^H NMR (400 MHz, CDCl_3_) δ 7.88 (2H, d, *J* = 6.9 Hz, Ph-H), 7.78 (1H, d, *J* = 12.4 Hz, CH=CH-NMe_2_), 7.39–7.45 (3H, m, Ph-H), 5.70 (1H, d, *J* = 12.4 Hz, CH=CH-NMe_2_), 3.11 (3H, s, N-CH_3_), 2.90 (3H, s, N-CH_3_). ^13^C NMR (100 MHz, CDCl_3_) δ 188.8, 154.4, 140.7, 131.0, 128.2 (2C), 127.6 (2C), 92.3, 45.1, 37.4. ESI MS: *m*/*z* 176 [M + H]^+^.

*(E)-3-(Dimethylamino)-1-(p-tolyl)prop-2-en-1-one* (**2b**). Yellow powder; m.p. 88–89 °C; yield, 0.62 g (24%); ^1^H NMR (400 MHz, CDCl_3_) δ 7.80 (2H, d, *J* = 8.1 Hz, Ph-H), 7.77 (1H, d, *J* = 12.3 Hz, CH=CH-NMe_2_), 7.19 (2H, d, *J* = 8.1 Hz, Ph-H), 5.70 (1H, d, *J* = 12.3 Hz, CH=CH-NMe_2_), 3.09 (3H, s, N-CH_3_), 2.89 (3H, s, N-CH_3_), 2.37 (3H, s, Ph-CH_3_). ^13^C NMR (100 MHz, CDCl_3_) δ 188.2, 153.9, 141.1, 137.7, 128.7 (2C), 127.5 (2C), 92.0, 44.8, 37.1, 21.3. ESI MS: *m*/*z* 190 [M + H]^+^.

*(E)-3-(Dimethylamino)-1-(2-hydroxyphenyl)prop-2-en-1-one* (**2d**). Orange powder; m.p. 133–135 °C; yield, 1.58 g (56%); ^1^H NMR (400 MHz, CDCl_3_) δ 13.98 (1H, s, C_2_-OH), 7.87 (1H, d, *J* = 12.3 Hz, CH=CH-NMe_2_), 7.69 (1H, dd, *J* = 8.1, 1.1 Hz, Ph-H), 7.35 (1H, m, Ph-H), 6.93 (1H, d, *J* = 8.1, Ph-H), 6.80 (1H, m, Ph-H), 5.77 (1H, d, *J* = 12.3 Hz, CH=CH-NMe_2_), 3.18 (3H, s, N-CH_3_), 2.96 (3H, s, N-CH_3_). ^13^C NMR (100 MHz, CDCl_3_) δ 191.6, 163.0, 154.9, 134.0, 128.3, 120.4, 118.3, 118.1, 90.1, 45.5, 37.5. ESI MS: *m*/*z* 192 [M + H]^+^.

*(E)-3-(Dimethylamino)-1-(pyridin-4-yl)prop-2-en-1-one* (**2e**). Yellow powder; m.p. 115–116 °C; yield, 0.886 g (34%); ^1^H NMR (400 MHz, CDCl_3_) δ 8.67 (2H, d, *J* = 5.6, Ar-H), 7.82 (1H, d, *J* = 11.9 Hz, CH=CH-NMe_2_), 7.67 (2H, d, *J* = 5.6 Hz, Ar-H), 5.63 (1H, d, *J* = 11.9 Hz, CH=CH-NMe_2_), 3.16 (3H, s, N-CH_3_), 2.93 (3H, s, N-CH_3_). ^13^C NMR (100 MHz, CDCl_3_) δ 186.4, 155.2, 149.9 (2C), 147.3, 121.2 (2C), 91.6, 45.2, 37.4. ESI MS: *m*/*z* 177 [M + H]^+^.

### 3.4. General Procedure for the Synthesis of Compounds **3a**–**r**

Method A (for **3a**–**g**, **3i**, **3m**–**r**): A mixture of pyridinium salt **1** (0.591 mmol), enaminone **2** (0.394 mmol), and sodium acetate (0.197 mmol) in isopropyl alcohol (3 mL) and water (1 mL) was placed into the microwave reactor and irradiated at 150 °C for 30 min. After cooling to room temperature, the solvent was then evaporated under reduced pressure. The products were isolated by column chromatography on silica gel, eluting with ethyl acetate-hexane mixture in different proportions.

Method B (for **3h**, **3k**):**A mixture of pyridinium salt **1** (0.591 mmol), enaminone **2** (0.394 mmol), and sodium acetate (0.197 mmol) in isopropyl alcohol (3 mL) and water (1 mL) was placed into the microwave reactor and irradiated at 150 °C for 30 min and left to cool to room temperature. After cooling, the precipitate was filtered-off and washed with ethanol, water (2 times) and ethanol again, then dried in air.

Method C (for **3j**, **3l**):**A mixture of pyridinium salt **1** (0.591 mmol), enaminone **2** (0.394 mmol), and sodium acetate (0.197 mmol) in isopropyl alcohol (3 mL) and water (1 mL) was placed into the microwave reactor and irradiated at 150 °C for 30 min and left to cool to room temperature. The reaction mixture was diluted with water (70 mL) and extracted with DCM. The combined organic layer was dried over Na_2_SO_4_. After filtration, the solvent was evaporated under reduced pressure. The residue was recrystallized from the isopropyl alcohol-DCM 3-1 mixture. The precipitate was filtered-off and washed with isopropyl alcohol for 3 times, then dried in air.

*2-Phenylpyrido[2,3-b]indolizine* (**3a**). Prepared according to the Method A. Eluent ethyl acetate-hexane 1: 10. Light brown powder; m.p. 178–180 °C (decomp.); yield, 48 mg (50%); ^1^H NMR (400 MHz, CDCl_3_) δ 8.25 (d, *J* = 7.1 Hz, 1H, H-6), 8.15 (d, *J* = 8.6 Hz, 1H, H-3), 8.11 (d, *J* = 7.6 Hz, 2H, Ph-H), 7.61 (d, *J* = 8.6 Hz, 1H, H-4), 7.50–7.52 (m, 3H, H-9, Ph-H), 7.42–7.44 (m, 1H, Ph-H), 6.95–6.98 (m, 1H, H-7), 6.90 (s, 1H, H-10), 6.53–6.55 (m, 1H, H-8). ^13^C NMR (100 MHz, CDCl_3_) δ 154.9, 146.3, 140.7, 139.2, 128.6 (2C), 128.4, 127.5 (2C), 124.6, 123.4, 121.7, 119.4, 118.0, 112.2, 108.7, 92.5. ESI MS: *m*/*z* 245 [M + H]^+^. Elemental analysis calcd (%) for C_17_H_12_N_2_: C 83.58; H 4.95; N 11.47; found: C 83.54; H 4.98; N 11.59.

*9-Methyl-2-phenylpyrido[2,3-b]indolizine* (**3b**). Prepared according to the Method A. Eluent ethyl acetate-hexane 1: 5. Yellow powder; m.p. 143–146 °C (decomp.); yield, 83 mg (82%); ^1^H NMR (400 MHz, CDCl_3_) δ 8.16–8.20 (m, 2H, H-3, H-6), 8.12 (d, *J* = 7.6 Hz, 2H, Ph-H), 7.62 (d, *J* = 8.6 Hz, 1H, H-4), 7.50–7.53 (m, 2H, Ph-H), 7.41–7.44 (m, 1H, m, Ph-H), 6.88 (s, 1H, H-10), 6.80 (d, *J* = 6.1 Hz, 1H, H-8), 6.52–6.55 (m, 1H, H-7), 2.51 (s, 3H, C_9_-CH_3_). ^13^C NMR (100 MHz, CDCl_3_) δ 154.7, 146.2, 140.6 (2C), 128.7 (3C), 128.4, 127.5 (2C), 122.3 (2C), 122.0, 118.3, 112.2, 109.0, 91.1, 18.5. ESI MS: *m*/*z* 259 [M + H]^+^. Elemental analysis calcd (%) for C_18_H_14_N_2_: C 83.69; H 5.46; N 10.84; found: C 83.66; H 5.51; N 10.95.

*7-Methyl-2-phenylpyrido[2,3-b]indolizine* (**3c**). Prepared according to the Method A. Eluent ethyl acetate-hexane 1: 5. Dark yellow powder; m.p. 175–176 °C (decomp.); yield, 27 mg (27%); ^1^H NMR (400 MHz, CDCl_3_) δ 8.15 (d, *J* = 8.6 Hz, 1H, H-3), 8.10 (d, *J* = 7.6 Hz, 2H, Ph-H), 8.08 (s, 1H, H-6), 7.59 (d, *J* = 8.6 Hz, 1H, H-4), 7.49–7.52 (m, 2H, Ph-H), 7.41–7.46 (m, 2H, H-9, Ph-H), 6.86–6.87 (m, 2H, H-8, H-10), 2.31 (3H, c, C_7_-CH_3_). ^13^C NMR (100 MHz, CDCl_3_) δ 154.5, 146.0, 140.7, 138.3, 128.6 (2C), 128.3, 127.5 (2C), 127.1, 121.8, 121.6, 119.0, 118.1 (2C), 112.0, 91.9, 18.3. ESI MS: *m*/*z* 259 [M + H]^+^. Elemental analysis calcd (%) for C_18_H_14_N_2_: C 83.69; H 5.46; N 10.84; found: C 83.62; H 5.48; N 10.99.

*2-(p-Tolyl)pyrido[2,3-b]indolizine* (**3d**). Prepared according to the Method A. Eluent ethyl acetate-hexane 1: 10. Bright yellow powder; m.p. 181–182 °C (decomp.); yield, 29 mg (29%); ^1^H NMR (400 MHz, CDCl_3_) δ 8.90 (d, *J* = 7.1 Hz, 1H, H-6), 8.67 (d, *J* = 8.6 Hz, 1H, H-3), 8.09 (d, *J* = 8.1 Hz, 2H, Ph-H), 7.80 (d, *J* = 8.6 Hz, 1H, H-4), 7.61 (d, *J* = 9.6 Hz, 1H, H-9), 7.30 (d, *J* = 8.1 Hz, 2H, Ph-H), 7.09–7.11 (m, 1H, H-7), 6.78 (s, 1H, H-10), 6.68–6.70 (m, 1H, H-8), 2.37 (s, 3H, Ph-CH_3_). ^13^C NMR (100 MHz, CDCl_3_) δ 153.2, 145.4, 138.7, 137.9, 137.2, 129.3 (2C), 126.8 (2C), 126.3, 124.1, 121.5, 119.6, 118.7, 111.3, 108.8, 91.5, 20.8. ESI MS: *m*/*z* 259 [M + H]^+^. Elemental analysis calcd (%) for C_18_H_14_N_2_: C, 83.69; H, 5.46; N, 10.84; found: C 83.65; H 5.49; N 10.96.

*9-Methyl-2-(p-tolyl)pyrido[2,3-b]indolizine* (**3e**). Prepared according to the Method A. Eluent ethyl acetate-hexane 1: 15. Yellow powder; m.p. 174 °C (decomp.); yield, 87 mg (81%); ^1^H NMR (400 MHz, CDCl_3_) δ 8.79 (d, *J* = 6.6 Hz, 1H, H-6), 8.66 (d, *J* = 8.6 Hz, 1H, H-3), 8.09 (d, *J* = 8.1 Hz, 2H, Ph-H), 7.80 (d, *J* = 8.6 Hz, 1H, H-4), 7.31 (d, *J* = 7.6 Hz, 2H, Ph-H), 6.93 (d, *J* = 6.1 Hz, 1H, H-8), 6.77 (s, 1H, H-10), 6.65–6.67 (m, 1H, H-7), 2.46 (s, 3H, C_9_-CH_3_), 2.37 (s, 3H, Ph-CH_3_). ^13^C NMR (100 MHz, CDCl_3_) δ 153.1, 145.4, 139.7, 137.8, 137.2, 129.2 (2C), 127.2, 126.8 (2C), 123.9, 122.5, 122.1, 119.8, 111.4, 108.9, 90.3, 20.8, 18.0. ESI MS: *m*/*z* 273 [M + H]^+^. Elemental analysis calcd (%) for C_19_H_16_N_2_: C, 83.79; H, 5.92; N, 10.29; found: C 83.72; H 5.89; N 10.43.

*7-Methyl-2-(p-tolyl)pyrido[2,3-b]indolizine* (**3f**). Prepared according to the Method A. Eluent ethyl acetate-hexane 1: 15. Dark yellow powder; m.p. 173–174 °C (decomp.); yield, 23 mg (21%); ^1^H NMR (400 MHz, CDCl_3_) δ 8.73 (s, 1H, H-6), 8.60 (d, *J* = 8.6 Hz, 1H, H-3), 8.08 (d, *J* = 8.1 Hz, 2H, Ph-H), 7.77 (d, *J* = 8.6 Hz, 1H, H-4), 7.55 (d, *J* = 9.3, 1H, H-9), 7.30 (d, *J* = 7.6 Hz, 2H, Ph-H), 6.98 (d, *J* = 9.3 Hz, 1H, H-8), 6.73 (s, 1H, H-10), 2.37 (s, 3H, Ph-CH_3_), 2.28 (s, 3H, C_7_-CH_3_). ^13^C NMR (100 MHz, CDCl_3_) δ 152.9, 145.3, 137.8, 137.6, 137.2, 129.2 (2C), 127.5, 126.7 (2C), 123.2, 121.3, 119.4, 118.3, 117.7, 111.1, 91.0, 20.8, 17.7. ESI MS: *m*/*z* 273 [M + H]^+^. Elemental analysis calcd (%) for C_19_H_16_N_2_: C, 83.79; H, 5.92; N, 10.29; found: C 83.84; H 5.90; N 10.33.

*2-(4-Bromophenyl)pyrido[2,3-b]indolizine* (**3g**). Prepared according to the Method A. Eluent ethyl acetate-hexane 1: 15. Brown powder; m.p. 215–217 °C (decomp.); yield, 33 mg (26%); ^1^H NMR (400 MHz, CDCl_3_) δ 8.93 (d, *J* = 7.1 Hz, 1H, H-6), 8.72 (d, *J* = 9.1 Hz, 1H, H-3), 8.15 (d, *J* = 8.6 Hz, 2H, Ph-H), 7.86 (d, *J* = 9.1 Hz, 1H, H-4), 7.69 (d, *J* = 8.6 Hz, 2H, Ph-H), 7.62 (d, *J* = 9.1 Hz, 1H, H-9), 7.11–7.14 (m, 1H, H-8), 6.80 (s, 1H, H-10), 6.70–6.73 (m, 1H, H-7). ^13^C NMR (100 MHz, CDCl_3_) δ 151.9, 145.5, 139.1, 139.0, 131.6 (2C), 128.9 (2C), 126.4, 124.5, 122.1, 121.8, 119.9, 118.7, 111.3, 108.9, 91.6. ESI MS: *m*/*z* 324 [M + H]^+^. Elemental analysis calcd (%) for C_17_H_11_BrN_2_: C 63.18; H 3.43; N 8.67; found: C 63.25; H 3.40; N 8.63.

*2-(4-Bromophenyl)-9-methylpyrido[2,3-b]indolizine* (**3h**). Prepared according to the Method B. Gold powder; m.p. 192–193 °C (decomp.); yield, 82 mg (62%); ^1^H NMR (400 MHz, CDCl_3_) δ 8.80 (d, *J* = 7.1 Hz, 1H, H-6), 8.70 (d, *J* = 8.6 Hz, 1H, H-3), 8.16 (d, *J* = 8.3 Hz, 2H, Ph-H), 7.85 (d, *J* = 8.6 Hz, 1H, H-4), 7.69 (d, *J* = 8.3 Hz, 2H, Ph-H), 6.95 (d, *J* = 6.6 Hz, 1H, H-8), 6.79 (s, 1H, H-10), 6.66–6.69 (m, 1H, H-7), 2.46 (s, 3H, C_9_-CH_3_). ^13^C NMR (100 MHz, CDCl_3_) δ 151.8, 145.5, 140.0, 139.1, 131.5 (2C), 128.9 (2C), 127.2, 124.0, 122.7, 122.4, 122.0, 119.9, 111.4, 109.0, 90.4, 18.0. ESI MS: *m*/*z* 338 [M + H]^+^. Elemental analysis calcd (%) for C_18_H_13_BrN_2_: C 64.11; H 3.89; N 8.31; found: C 64.01; H 3.91; N 8.36.

*2-(4-Bromophenyl)-7-methylpyrido[2,3-b]indolizine* (**3i**). Prepared according to the Method A. Eluent ethyl acetate-hexane 1: 15. Light brown powder; m.p. 177–178 °C (decomp.); yield, 25 mg (19%); ^1^H NMR (400 MHz, CDCl_3_) δ 8.72 (s, 1H, H-6), 8.61 (d, *J* = 8.1 Hz, 1H, H-3), 8.14 (d, *J* = 7.6 Hz, 2H, Ph-H), 7.79 (d, *J* = 8.1 Hz, 1H, H-4), 7.67 (d, *J* = 7.6 Hz, 2H, Ph-H), 7.55 (d, *J* = 9.1 Hz, 1H, H-9), 6.99 (d, *J* = 9.1 Hz, 1H, H-8), 6.74 (s, 1H, H-10), 2.27 (s, 3H, C_7_-CH_3_). ^13^C NMR (100 MHz, CDCl_3_) δ 151.5, 145.3, 139.1, 137.9, 131.5 (2C), 128.9 (2C), 127.7, 123.2, 121.9, 121.5, 119.5, 118.3, 117.9, 111.1, 91.1, 17.7. ESI MS: *m*/*z* 338 [M + H]^+^. Elemental analysis calcd (%) for C_18_H_13_BrN_2_: C 64.11; H 3.89; N 8.31; found: C 64.17; H 3.92; N 8.34.

*2-(Pyrido[2,3-b]indolizin-2-yl)phenol* (**3j**). Prepared according to the Method C. Dark brown powder; m.p. 230–232 °C (decomp.); yield, 38 mg (37%); ^1^H NMR (400 MHz, CDCl_3_) δ 15.09 (s, 1H, OH), 8.99 (d, *J* = 6.6 Hz, 1H, H-6), 8.85 (d, *J* = 8.9 Hz, 1H, H-3), 8.13 (d, *J* = 7.6 Hz, 1H, Ph-H), 8.06 (d, *J* = 8.9 Hz, 1H, H-4), 7.65 (d, *J* = 9.1 Hz, 1H, H-9), 7.29–7.31 (m, 1H, Ph-H), 7.18–7.20 (m, 1H, H-8), 6.92–6.96 (m, 2H, Ph-H), 6.84 (s, 1H, H-10), 6.78–6.80 (m, 1H, H-7). ^13^C NMR (100 MHz, CDCl_3_) δ 159.5, 154.4, 141.6, 139.3, 130.8, 127.3, 126.3, 125.1, 121.6, 121.5, 119.7, 118.7, 118.6, 117.8, 110.2, 109.5, 90.3. ESI MS: *m*/*z* 261 [M + H]^+^. Elemental analysis calcd (%) for C_17_H_12_N_2_O: C 78.44; H 4.65; N 10.76; found: C 78.39; H 4.62; N 10.82.

*2-(9-Methylpyrido[2,3-b]indolizin-2-yl)phenol* (**3k**). Prepared according to the Method B. Gold powder; m.p. 179 °C (decomp.); yield, 71 mg (66%); ^1^H NMR (400 MHz, CDCl_3_) δ 15.10 (s, 1H, OH), 8.87 (d, *J* = 6.6 Hz, 1H, H-6), 8.84 (d, *J* = 8.6 Hz, 1H, H-3), 8.13 (d, *J* = 7.6 Hz, 1H, Ph-H), 8.06 (d, *J* = 8.6 Hz, 1H, H-4), 7.29–7.31 (m, 1H, Ph-H), 7.01 (d, *J* = 6.6 Hz, 1H, H-8), 6.92–6.96 (m, 2H, Ph-H), 6.84 (s, 1H, H-10), 6.73–6.76 (m, 1H, H-7), 2.47 (s, 3H, C_9_-CH_3_). ^13^C NMR (100 MHz, CDCl_3_) δ 159.5, 154.3, 141.5, 140.3, 130.8, 127.3, 127.2, 123.9, 123.4, 122.1, 121.6, 119.7, 118.6, 117.9, 110.3, 109.6, 89.2, 17.9. ESI MS: *m*/*z* 275 [M + H]^+^. Elemental analysis calcd (%) for C_18_H_14_N_2_O: C 78.81; H 5.14; N 10.21; found: C 78.77; H 5.16; N 10.30.

*2-(7-Methylpyrido[2,3-b]indolizin-2-yl)phenol* (**3l**). Prepared according to the Method C. Brown powder; m.p. 226–227 °C (decomp.); yield, 26 mg (24%); ^1^H NMR (400 MHz, CDCl_3_) δ 15.13 (s, 1H, OH), 8.81 (s, 1H, H-6), 8.78 (d, *J* = 8.9 Hz, 1H, H-3), 8.11 (d, *J* = 7.1 Hz, 1H, Ph-H), 8.03 (d, *J* = 8.9 Hz, 1H, H-4), 7.60 (d, *J* = 9.1 Hz, 1H, H-9), 7.28–7.30 (m, 1H, Ph-H), 7.07 (d, *J* = 9.1 Hz, 1H, H-8), 6.91–6.95 (m, 2H, Ph-H), 6.78 (s, 1H, H-10), 2.30 (s, 3H, C_9_-CH_3_). ^13^C NMR (100 MHz, CDCl_3_) δ 159.5, 154.1, 141.4, 138.3, 130.7, 128.4, 127.2, 123.2, 121.3, 121.2, 119.7, 118.6 (2C), 118.3, 117.8, 110.0, 89.8, 17.7. ESI MS: *m*/*z* 275 [M + H]^+^. Elemental analysis calcd (%) for C_18_H_14_N_2_O: C 78.81; H 5.14; N 10.21; found: C 78.83; H 5.11; N 10.28.

*2-(Pyridin-4-yl)pyrido[2,3-b]indolizine* (**3m**). Prepared according to the Method A. Eluent ethyl acetate-hexane 1: 5. Orange powder; m.p. 196–199 °C (decomp.); yield, 30 mg (31%); ^1^H NMR (400 MHz, CDCl_3_) δ 8.73 (d, *J* = 5.9 Hz, 2H, Py-H), 8.29 (d, *J* = 6.6 Hz, 1H, H-6), 8.21 (d, *J* = 8.6 Hz, 1H, H-3), 8.01 (d, *J* = 5.9 Hz, 2H, Py-H), 7.66 (d, *J* = 8.6 Hz, 1H, H-4), 7.52 (d, *J* = 9.6 Hz, 1H, H-9), 7.00–7.03 (m, 1H, H-7), 6.90 (s, 1H, H-10), 6.58–6.60 (m, 1H, H-8). ^13^C NMR (100 MHz, CDCl_3_) δ 151.7, 150.2 (2C), 147.7, 146.5, 139.9, 124.7, 124.0, 122.4, 121.7 (2C), 119.6, 118.2, 111.8, 109.2, 92.7. ESI MS: *m*/*z* 246 [M + H]^+^. Elemental analysis calcd (%) for C_16_H_11_N_3_: C 78.35; H 4.52; N 17.13; found: C 78.31; H 4.55; N 17.20.

*9-Methyl-2-(pyridin-4-yl)pyrido[2,3-b]indolizine* (**3n**). Prepared according to the Method A. Eluent ethyl acetate-hexane 1: 5. Light beige powder; m.p. 173–174 °C (decomp.); yield, 34 mg (33%); ^1^H NMR (400 MHz, CDCl_3_) δ 8.72 (d, *J* = 5.8 Hz, 1H, Py-H), 8.18–8.20 (m, 2H, H-3, H-6), 8.01 (d, *J* = 5.8 Hz, 2H, Py-H), 7.65 (d, *J* = 8.6 Hz, 1H, H-4), 6.86 (s, 1H, H-10), 6.82 (d, *J* = 6.6 Hz, 1H, H-8), 6.54–6.56 (m, 1H, H-7), 3.18 (s, 3H, C_9_-CH_3_). ^13^C NMR (100 MHz, CDCl_3_) δ 151.5, 150.5, 150.2 (2C), 147.7, 141.1, 128.5, 123.6, 122.5, 122.3, 121.6 (2C), 118.3, 111.8, 109.3, 91.2, 18.5. ESI MS: *m*/*z* 260 [M + H]^+^. Elemental analysis calcd (%) for C_17_H_13_N_3_: C 78.74; H 5.05; N 16.20; found: C 78.71; H 5.09; N 16.29.

*7-Methyl-2-(pyridin-4-yl)pyrido[2,3-b]indolizine* (**3o**). Prepared according to the Method A. Eluent ethyl acetate-hexane 1: 5. Light beige powder; m.p. 223–225 °C (decomp.); yield, 27 mg (26%); ^1^H NMR (400 MHz, CDCl_3_) δ 8.73 (d, *J* = 5.8 Hz, 2H, Py-H), 8.21 (d, *J* = 8.6 Hz, 1H, H-3), 8.11 (s, 1H, H-6), 8.03 (d, *J* = 5.8 Hz, 2H, Py-H), 7.66 (d, *J* = 8.6 Hz, 1H, H-4), 7.47 (d, *J* = 8.9 Hz, 1H, H-9), 6.90 (d, *J* = 8.9 Hz, 1H, H-8), 6.87 (s, 1H, H-10), 2.33 (s, 3H, C_7_-CH_3_). ^13^C NMR (100 MHz, CDCl_3_) δ 151.3, 150.1 (2C), 147.9, 146.4, 139.0, 127.6, 122.2, 121.9, 121.7 (2C), 119.1, 118.6, 118.1, 111.6, 92.2, 18.3. ESI MS: *m*/*z* 260 [M + H]^+^. Elemental analysis calcd (%) for C_17_H_13_N_3_: C 78.74; H 5.05; N 16.20; found: C 78.69; H 5.06; N 16.32.

*2-(Pyridin-2-yl)pyrido[2,3-b]indolizine* (**3p**). Prepared according to the Method A. Eluent ethyl acetate-hexane 1: 10. Yellow powder; m.p. 159–162 °C (decomp.); yield, 27 mg (28%); ^1^H NMR (400 MHz, CDCl_3_) δ 8.72 (d, *J* = 4.0 Hz, 1H, Pyr-H), 8.60 (d, *J* = 8.1 Hz, 1H, Py-H), 8.38 (d, *J* = 8.6 Hz, 1H, H-3), 8.33 (d, *J* = 7.1 Hz, 1H, H-6), 8.28 (d, *J* = 8.6 Hz, 1H, H-4), 7.86 (m, 1H, Py-H), 7.52 (d, *J* = 9.1 Hz, 1H, H-9), 7.31–7.33 (m, 1H, Py-H), 6.99–7.01 (m, 1H, H-7), 6.91 (s, 1H, H-10), 6.58–6.60 (1H, m, H-8), 6.58–6.60 (m, 1H, H-8). ^13^C NMR (100 MHz, CDCl_3_) δ 157.2, 153.4, 149.0, 145.9, 139.5, 136.9, 124.7, 123.6, 123.3, 122.8, 121.5, 119.5, 118.2, 112.5, 108.9, 92.5. ESI MS: *m*/*z* 246 [M + H]^+^. Elemental analysis calcd (%) for C_16_H_11_N_3_: C 78.35; H 4.52; N 17.13; found: C 78.32; H 4.57; N 17.14.

*9-Methyl-2-(pyridin-2-yl)pyrido[2,3-b]indolizine* (**3q**). Prepared according to the Method A. Eluent ethyl acetate-hexane 1: 10. Lime-green powder; m.p. 124–127 °C (decomp.); yield, 71 mg (70%); ^1^H NMR (400 MHz, CDCl_3_) δ 8.71 (d, *J* = 4.0 Hz, 1H, Py-H), 8.61 (d, *J* = 7.6 Hz, 1H, Py-H), 8.37 (d, *J* = 8.6 Hz, 1H, H-3), 8.25 (d, *J* = 8.6 Hz, 1H, H-4), 8.22 (d, *J* = 7.1 Hz, 1H, H-6), 7.84–7.87 (m, 1H, Py-H), 7.30–7.32 (m, 1H, Py-H), 6.88 (s, 1H, H-10), 6.80 (d, *J* = 6.1 Hz, 1H, H-8), 6.53–6.55 (m, 1H, H-7), 2.51 (s, 3H, C_9_-CH_3_). ^13^C NMR (100 MHz, CDCl_3_) δ 157.2, 153.3, 149.0, 145.9, 140.7, 136.8, 128.4, 123.4, 123.2, 122.4, 122.1, 121.5, 118.3, 112.5, 109.1, 91.1, 18.5. ESI MS: *m*/*z* 260 [M + H]^+^. Elemental analysis calcd (%) for C_17_H_13_N_3_: C 78.74; H 5.05; N 16.20; found: C 78.70; H 5.07; N 16.25.

*7-Methyl-2-(pyridin-4-yl)pyrido[2,3-b]indolizine* (**3r**). Prepared according to the Method A. Eluent ethyl acetate-hexane 1: 7. Brown powder; m.p. 74–79 °C (decomp.); yield, 39 mg (38%); ^1^H NMR (400 MHz, CDCl_3_) δ 8.71 (d, *J* = 4.0 Hz, 1H, Py-H), 8.59 (d, *J* = 8.1 Hz, 1H, Py-H), 8.35 (d, *J* = 8.6 Hz, 1H, H-3), 8.25 (d, *J* = 8.6 Hz, 1H, H-4), 8.13 (s, 1H, H-6), 7.83–7.86 (m, 1H, Py-H), 7.47 (d, *J* = 9.1 Hz, 1H, H-9), 7.30–7.32 (m, 1H, Py-H), 6.86–6.89 (m, 2H, H-8, H-10), 2.33 (s, 3H, C_7_-CH_3_). ^13^C NMR (100 MHz, CDCl_3_) δ 157.4, 153.2, 149.0, 146.0, 138.5, 136.8, 127.1, 123.2, 122.6, 121.9, 121.5, 119.0, 118.2, 118.0, 112.3, 92.1, 18.3. ESI MS: *m*/*z* 260 [M + H]^+^. Elemental analysis calcd (%) for C_17_H_13_N_3_: C 78.74; H 5.05; N 16.20; found: C 78.71; H 5.09; N 16.33.

## 4. Conclusions

In conclusion, we discovered a novel domino route to condensed indolizines—pyrido[2,3-*b*]indolizines, containing various aromatic or heteroaromatic moieties at C(2) and alkyl groups at C(7) or C(9). The route is based on the interaction of 2-alkyl-*N*-(cyanomethyl)pyridinium salts with enaminones. The synthesized compounds are effective fluorophores, emitting green light with FQYs up to 82%.

## Figures and Tables

**Table 1:**
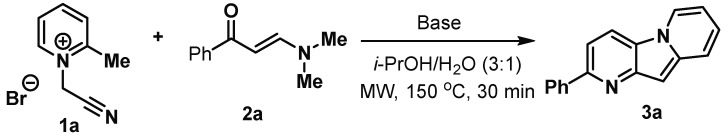
Reaction conditions optimization.

Entry ^a^	Base	1a: 2a: Base	Yield, % ^b^
1	NaOAc	3: 1: 5	34
2		3: 1: 1	40
3		3: 1: 0.5	46
4		3: 1: 0.1	12
5		1.5: 1: 0.5	50
6		1: 1: 0.5	44
7	Et_3_N	1.5: 1: 0.5	31
8	DIPEA	1.5: 1: 0.5	25
9	NH_4_OAc	1.5: 1: 0.5	34
10	K_2_CO_3_	1.5: 1: 0.5	5
11	Cs_2_CO_3_	1.5: 1: 0.5	21
12 ^c^	NaOAc	1.5: 1: 0.5	47
13 ^d^	NaOAc	1.5: 1: 0.5	33
14 ^e^	NaOAc	1.5: 1: 0.5	37

^a^ A mixture of pyridinium salt **1** (0.591 mmol), enaminone **2** and the corresponding base in isopropyl alcohol (3 mL) and water (1 mL) was irradiated in a closed vessel in a microwave reactor Monowave 300 (Anton Paar GmbH) at 150 °C for 30 min. ^b^ Isolated yield. ^c^ The reaction time was prolonged from 30 to 60 min. ^d^ The reaction temperature was 120 °C. ^e^ The reaction temperature was 180 °C.

**Table 2:** Photophysical properties of the synthesized compound.

Compound	Abs ^[a]^	ε ^[b]^	Emission ^[a]^	FQY ^[c]^	Stokes Shift
	[nm]	[(M cm)^−1^ (10^9^)]	[nm]	[%]	[cm^−1^]
**3a**	413	1.652	511	77	4643
**3b**	404	1.616	505	82	4950
**3c**	414	1.656	520	64	4923
**3m**	418	1.672	519	57	4655
**3n**	410	1.640	513	63	4897
**3o**	420	1.680	528	55	4870
**3p**	416	1.664	516	64	4658
**3q**	409	1.636	512	77	4918

^[a]^ Peak maximum. ^[b]^ Molar extinction coefficient. ^[c]^ Fluorescence quantum yield.

## Supplementary Materials

The supplementary materials are available online.

## References

[1] Kappert F., Sreeramulu S., Jonker H.R.A., Richter C., Rogov V.V., Proschak E., Hargittay B., Saxena K., Schwalbe H. (2018). Structural characterization of the interaction of the fibroblast growth factor receptor with a small molecule allosteric inhibitor. Chemistry.

[2] Yu Q., Yang H., Zhu T., Yu L., Chen J., Gu L., Huang Z., An L. (2018). Synthesis, cytotoxicity and structure-activity relationship of indolizinoquinolinedione derivatives as DNA topoisomerase IB catalytic inhibitors. Eur. J. Med. Chem..

[3] Park S., Kim E.H., Kim J., Kim S.H., Kim I. (2018). Biological evaluation of indolizine-chalcone hybrids as new anticancer agents. Eur. J. Med. Chem..

[4] Tatipamula V.B., Kolli M.K., Lagu S.B., Paidi K.R., Rsddy R., Yejella R.P. (2019). Novel indolizine derivatives lowers blood glucose levels in streptozotocin-induced diabetic rats: A histopathological approach. Pharm. Rep..

[5] Moon S.H., Jung Y., Kim S.H., Kim I. (2016). Synthesis, characterization and biological evaluation of anti-cancer indolizine derivatives via inhibiting β-catenin activity and activating p53. Bioorg. Med. Chem. Lett..

[6] Sardaru M.C., Craciun A.M., Al Matarneh C.M., Sandu I.A., Amarandi R.M., Popovici L., Ciobanu C.I., Peptanariu D., Pinteala M., Mangalagiu I.I. (2020). Cytotoxic substituted indolizines as new colchicine site tubulin polymerisation inhibitors. J. Enzym. Inhib. Med. Chem..

[7] Albaladejo M.J., González-Soria M.J., Alonso F. (2018). Metal-free remote-site C-H alkenylation: Regio- and diastereoselective synthesis of solvatochromic dyes. Green Chem..

[8] Gayton J., Autry S.A., Meador W., Parkin S.R., Hill G.A., Hammer N.I., Delcamp J.H. (2019). Indolizine-Cyanine dyes: Near infrared emissive cyanine dyes with increased Stokes shifts. J. Org. Chem..

[9] Cheema H., Baumann A., Loya E.K., Brogdon P., McNamara L.E., Carpenter C.A., Hammer N.I., Mathew S., Risko C., Delcamp J.H. (2019). Near-Infrared-Absorbing indolizine-porphyrin push-pull dye for dye-sensitized solar cells. ACS Appl. Mater. Interfaces.

[10] Yang J., Zhu Y., Tse A.K.W., Zhou X., Chen Y., Tse Y.C., Wong K.M.C., Ho C.Y. (2019). Synthesis and study of Au(iii)-indolizine derivatives: Turn-on luminescence by photo-induced controlled release. Chem. Commun..

[11] Lee Y., Cho W., Sung J., Kim E., Park S.B. (2018). Monochromophoric design strategy for tetrazine-based colorful bioorthogonal probes with a single fluorescent core skeleton. J. Am. Chem. Soc..

[12] Ji R., Liu A., Shen S., Cao X., Li F., Ge Y. (2017). An indolizine-rhodamine based FRET fluorescence sensor for highly sensitive and selective detection of Hg^2+^ in living cells. RSC Adv..

[13] Bayazit M.K., Pålsson L.O., Coleman K.S. (2015). Sensing properties of light-emitting single walled carbon nanotubes prepared via click chemistry of ylides bound to the nanotube surface. RSC Adv..

[14] Airinei A., Tigoianu R., Danac R., Al Matarneh C.M., Isac D.L. (2018). Steady state and time resolved fluorescence studies of new indolizine derivatives with phenanthroline skeleton. J. Lumin..

[15] Kucukdisli M., Opatz T. (2012). A modular synthesis of polysubstituted indolizines. Eur. J. Org. Chem..

[16] Cai Q., Zhu Y.P., Gao Y., Sun J.J., Wu A.X. (2013). A direct method for the synthesis of indolizine derivatives from easily available aromatic ketones, pyridines, and acrylonitrile derivatives. Can. J. Chem..

[17] Wang W., Han J., Sun J., Liu Y. (2017). CuBr-Catalyzed aerobic decarboxylative cycloaddition for the synthesis of indolizines under solvent-free conditions. J. Org. Chem..

[18] Wang D., Zhang X., He C., Duan C. (2010). Aminonaphthalimide-based imidazolium podands for turn-on fluo. Org. Biomol. Chem..

[19] Bonte S., Ghinea I.O., Dinica R., Baussanne I., Demeunynck M. (2016). Investigation of the pyridinium ylide-alkyne cycloaddition as a fluorogenic coupling reaction. Molecules.

[20] Yavari I., Ghafouri K., Naeimabadi M., Halvagar M.R. (2018). A synthesis of functionalized 2-Indolizin-3-yl-1,3-benzothiazoles from 1-(1,3-Benzothiazol-2-ylmethyl)pyridinium Iodide and Acetylenic Esters. Synlett.

[21] Yavari I., Sheykhahmadi J., Naeimabadi M., Halvagar M.R. (2017). Iodine-mediated sp^3^ C–H functionalization of methyl ketones: A one-pot synthesis of functionalized indolizines via the 1,3-dipolar cycloaddition reaction between pyridinium ylides and ynones. Mol. Divers..

[22] Douglas T., Pordea A., Dowden J. (2017). Iron-Catalyzed indolizine synthesis from pyridines, diazo compounds, and alkynes. Org. Lett..

[23] Shang Y., Zhang M., Yu S., Ju K., Wang C., He X. (2009). New route synthesis of indolizines via 1,3-dipolar cycloaddition of pyridiniums and alkynes. Tetrahedron Lett..

[24] Fu Q., Yan C.G. (2013). Molecular diversity of cycloaddition reactions of the functionalized pyridinium salts with 3-phenacylideneoxindoles. Tetrahedron.

[25] Zheng P., Li C., Mou C., Pan D., Wu S., Xue W., Jin Z., Chi Y.R. (2019). Efficient access to 2-Pyrones via carbene-catalyzed oxidative [3+3] reactions between enals and nitrogen Ylides. Asian J. Org. Chem..

[26] Zhang Y.F., Duan W.D., Chen J., Hu Y. (2019). Base-Promoted cascade reactions of 3-(1-Alkynyl)chromones with pyridinium Ylides to Chromeno[2,3-D]azepine derivatives. J. Org. Chem..

[27] Kucukdisli M., Opatz T. (2014). Two-step synthesis of 2-aminoindolizines from 2-alkylpyridines. Eur. J. Org. Chem..

[28] Storozhenko O.A., Festa A.A., Ndoutoume D.R.B., Aksenov A.V., Varlamov A.V., Voskressensky L.G. (2018). Mn-mediated sequential three-component domino Knoevenagel/cyclization/Michael addition/oxidative cyclization reaction towards annulated imidazo[1,2-a]pyridines. Beilstein J. Org. Chem..

[29] Voskressensky L.G., Storozhenko O.A., Festa A.A., Novikov R.A., Varlamov A.V. (2017). Synthesis of Chromenoimidazoles, annulated with an azaindole moiety, through a base-promoted domino reaction of cyano methyl quaternary salts. Synthesis.

[30] Voskressensky L.G., Sokolova E.A., Festa A.A., Varlamov A.V. (2013). A novel domino condensation-intramolecular nucleophilic cyclization approach towards annulated thiochromenes. Tetrahedron Lett..

[31] Sokolova E.A., Festa A.A., Golantsov N.E., Lukonina N.S., Ioffe I.N., Varlamov A.V., Voskressensky L.G. (2019). Highly fluorescent pyrido[2,3-b]indolizine-10-carbonitriles through pseudo three-component reactions of *N*-(Cyanomethyl)pyridinium salts. Eur. J. Org. Chem..

[32] Kro R., Mostafavi M., Lampre I. (2002). Preferential solvation of coumarin 153. The role of hydrogen bonding. J. Phys. Chem. A.

[33] Shen Y.M., Grampp G., Leesakul N., Hu H.W., Xu J.H. (2007). Synthesis and emitting properties of the blue-light fluorophores indolizino[3,4,5-ab]isoindole derivatives. Eur. J. Org. Chem..

[34] Park S., Kwon D.I., Lee J., Kim I. (2015). When indolizine meets quinoline: Diversity-oriented synthesis of new polyheterocycles and their optical properties. ACS Comb. Sci..

[35] Singh D.K., Kim S., Lee J.H., Lee N.K., Kim J., Lee J., Kim I. (2020). 6-(Hetero)arylindolizino[1,2-c]quinolines as highly fluorescent chemical space: Synthesis and photophysical properties. J. Heterocycl. Chem..

[36] Sung J., Lee Y., Cha J.H., Park S.B., Kim E. (2017). Development of fluorescent mitochondria probe based on 1,2-dihydropyrrolo[3,4-b]indolizine-3-one. Dyes Pigments.

[37] Al-Zaydi K.M., Borik R.M. (2007). Microwave assisted condensation reactions of 2-aryl hydrazonopropanals with nucleophilic reagents and dimethyl acetylenedicarboxylate. Molecules.

