# Supplementary material for: Microwave-Assisted Synthesis of Fluorescent Pyrido[2,3-b]indolizines from Alkylpyridinium Salts and Enaminones
Source: Molecules. 2020 Sep 5;25(18):4059. doi: 10.3390/molecules25184059 (PMC7570714; doi:10.3390/molecules25184059)
Supplement: Supplementary file 1 [file molecules-25-04059-s001.pdf]

Microwave-Assisted Synthesis of Fluorescent Pyrido[2,3-*b*]indolizines from Alkylpyridinium Salts and Enaminones

Ekaterina A. Sokolova [1], Alexey A. Festa [1], Karthikeyan Subramani [1], Victor B. Rybakov [2], Alexey V. Varlamov [1], Leonid G. Voskressensky [1], and Erik V. Van der Eycken [1,3,*]

[1] Organic Chemistry Department, Science Faculty, RUDN University, Moscow, Russian Federation, Miklukho-Maklaya st., 6;

[2] Department of Chemistry, Lomonosov Moscow State University, Leninskie Gory, 1-3, 119991, Moscow (Russia);

[3] Laboratory for Organic & Microwave-Assisted Chemistry (LOMAC), Department of Chemistry, University of Leuven (KU Leuven), Celestijnenlaan 200F, B-3001, Leuven, Belgium; erik.vandereycken@kuleuven.be

* Correspondence: erik.vandereycken@kuleuven.be;

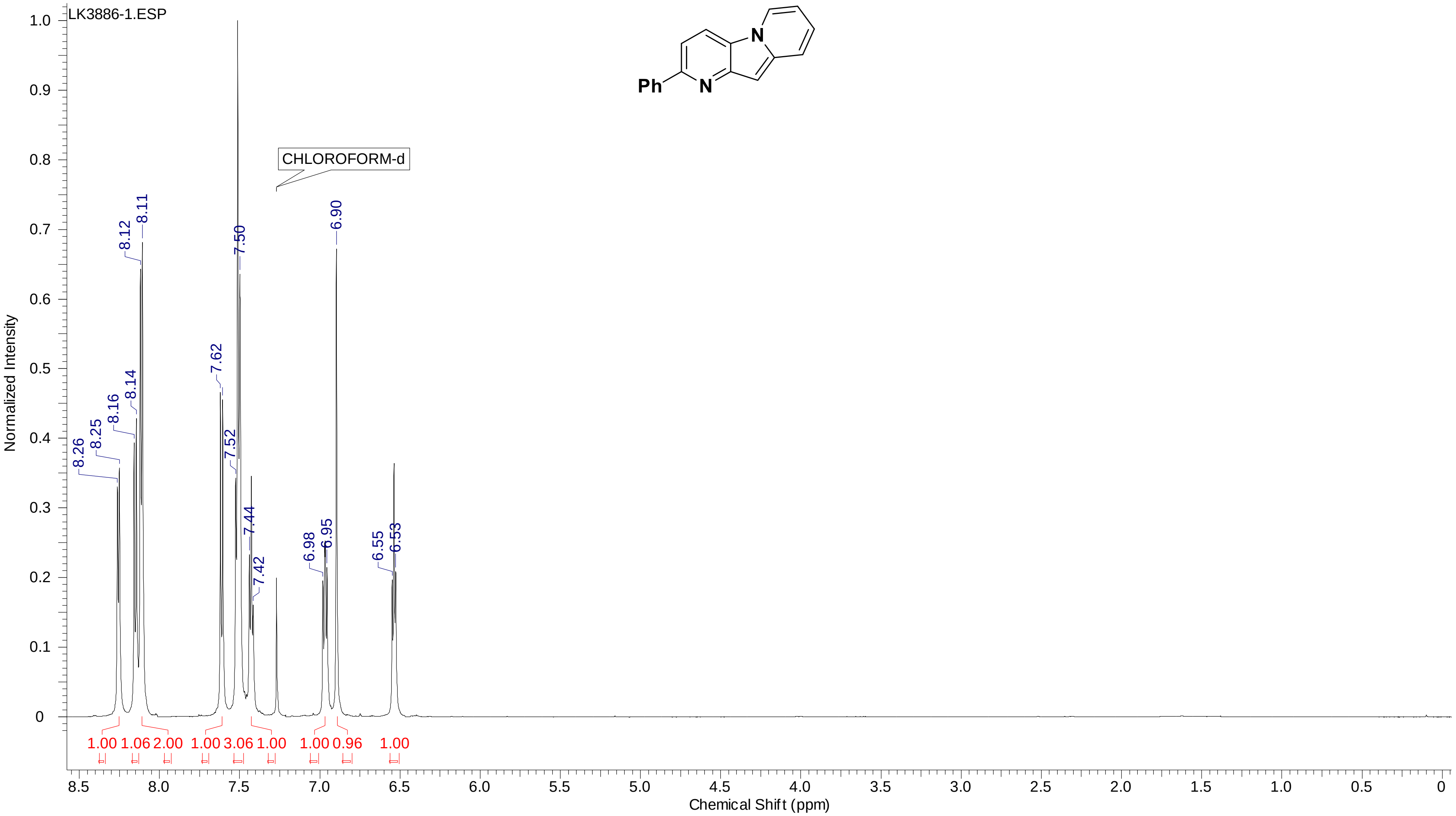

^{1}H NMR of compound **3a**

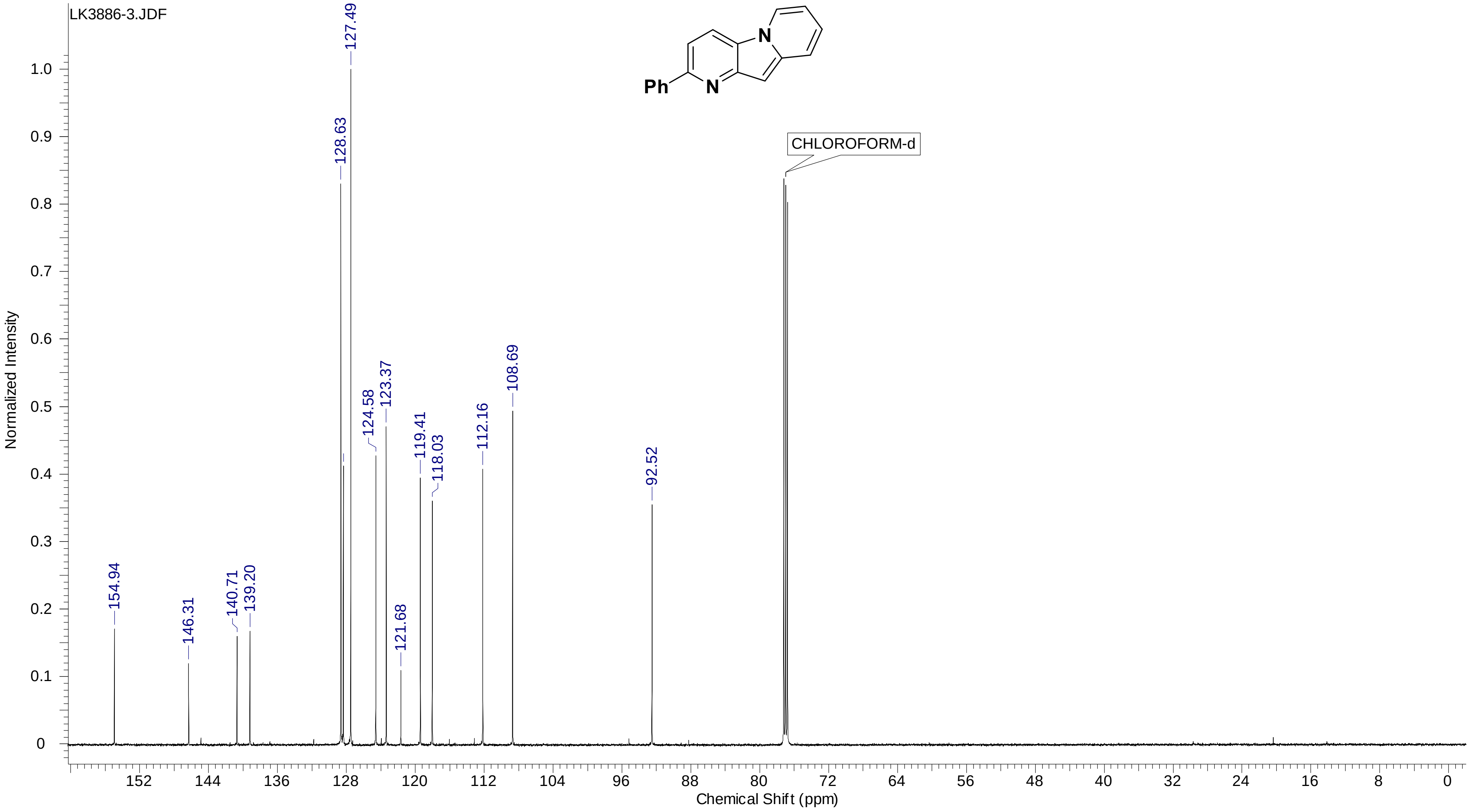

^{13}C NMR of compound **3a**

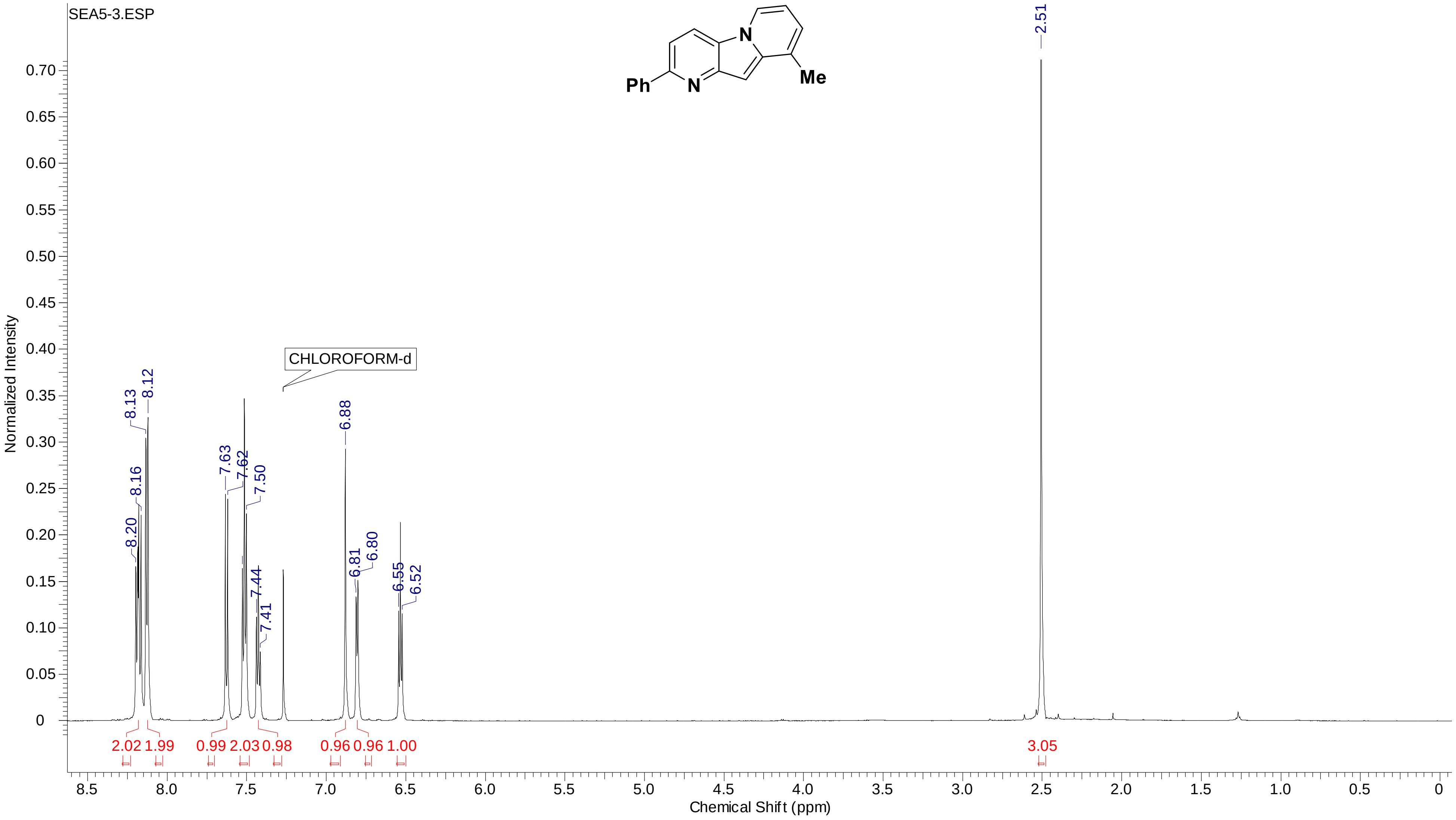

^{1}H NMR of compound **3b**

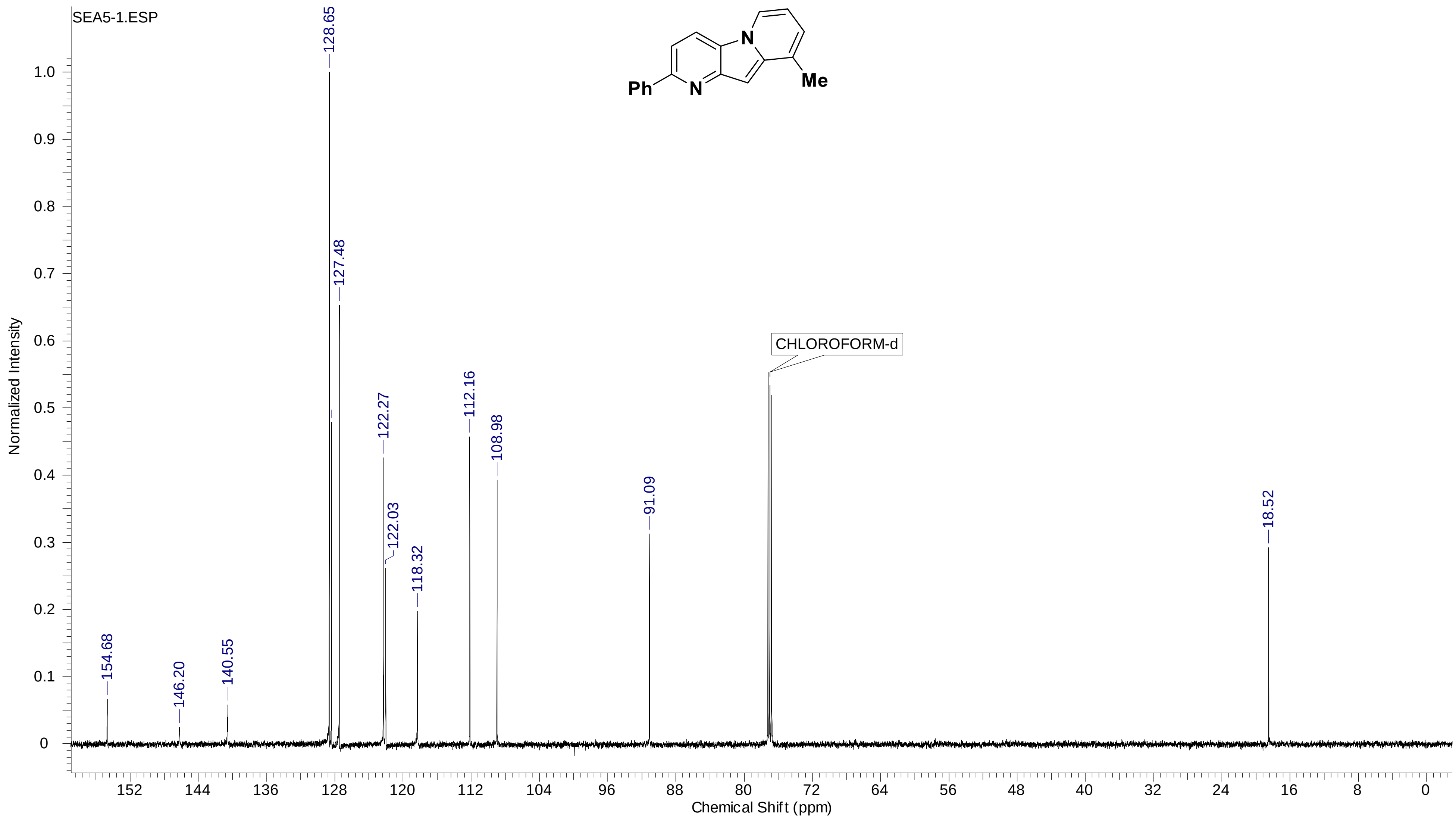

^{13}C NMR of compound **3b**

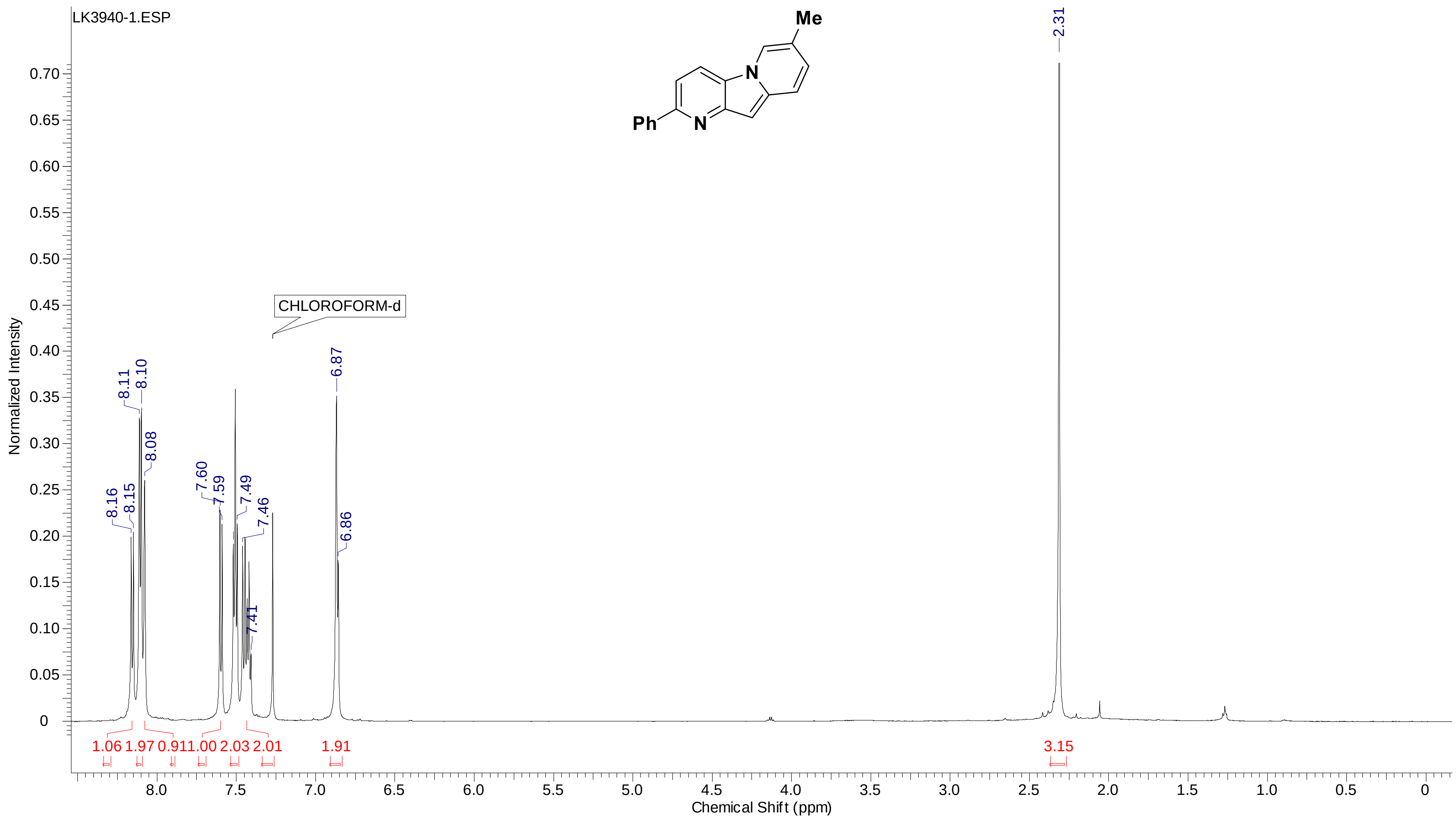

^{1}H NMR of compound **3c**

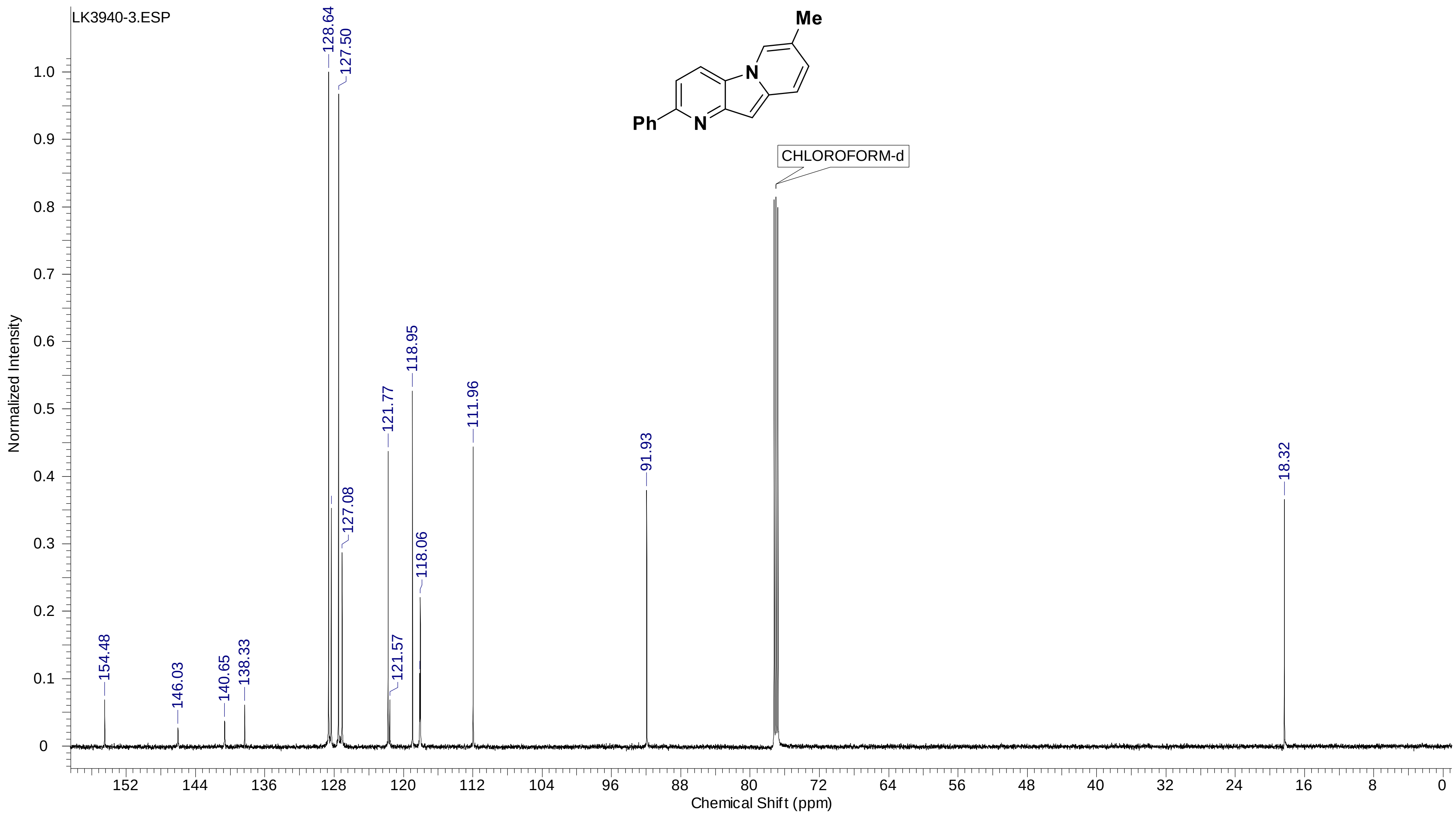

^{13}C NMR of compound **3c**

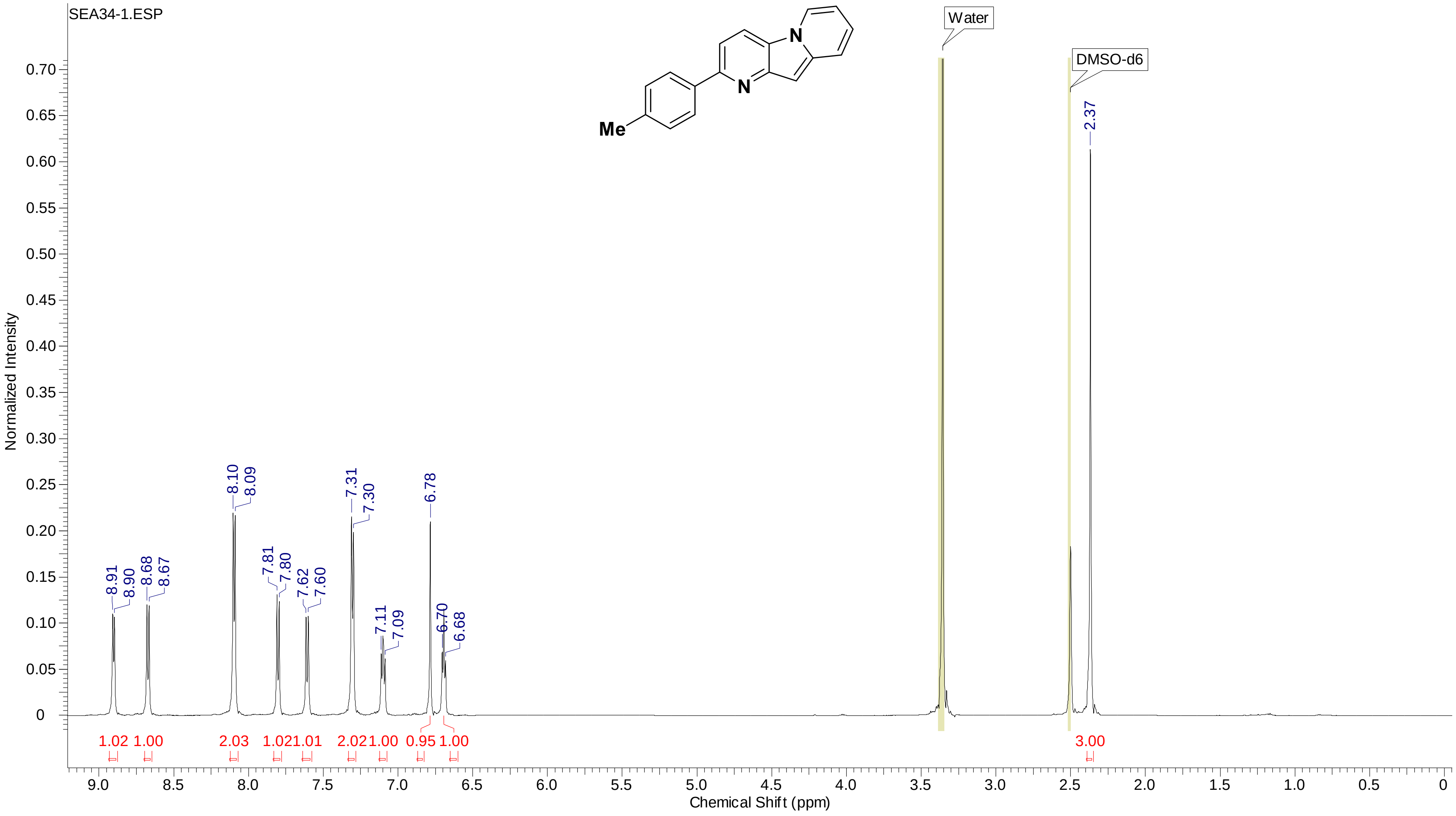

^{1}H NMR of compound **3d**

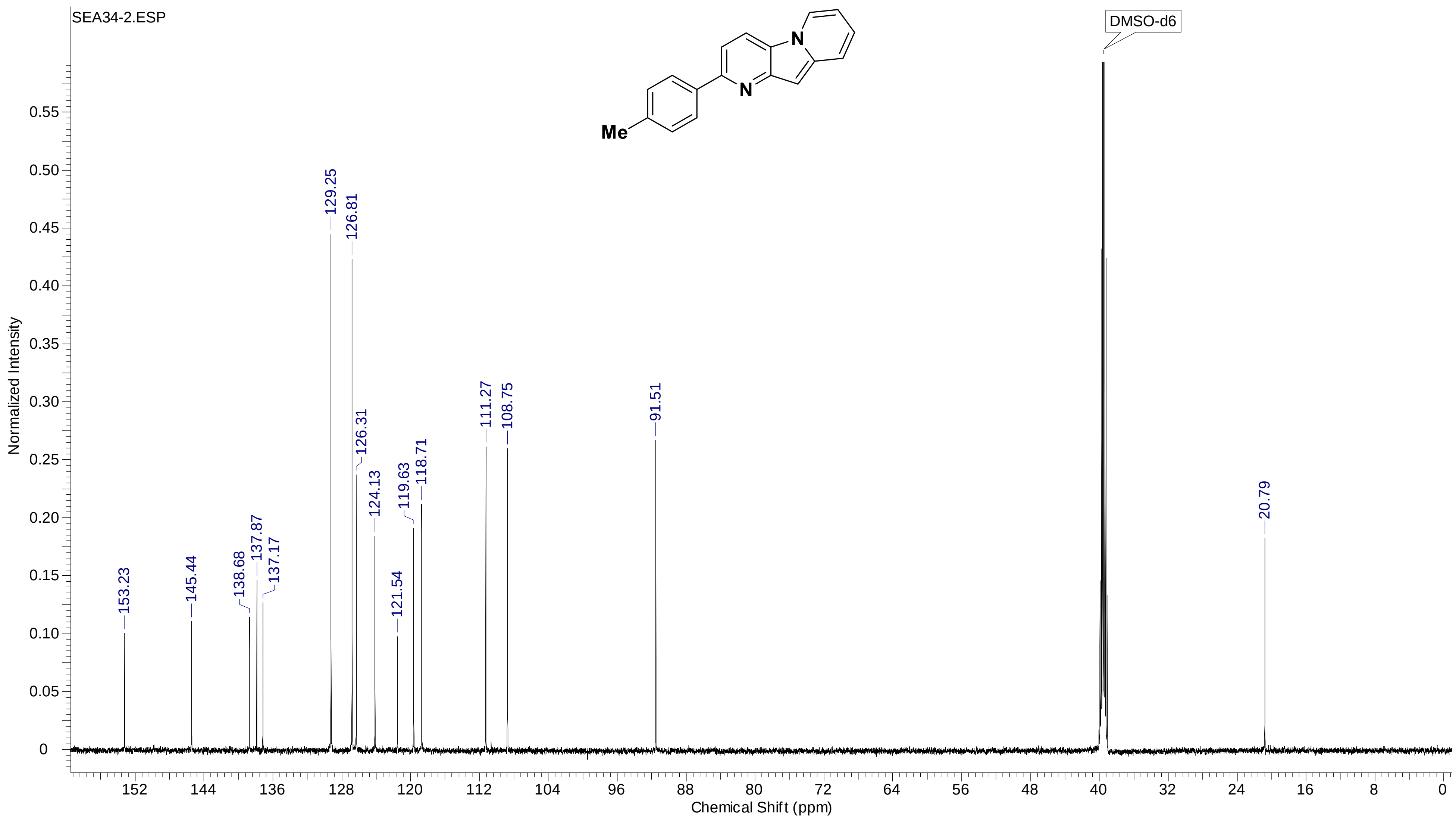

^{13}C NMR of compound **3d**

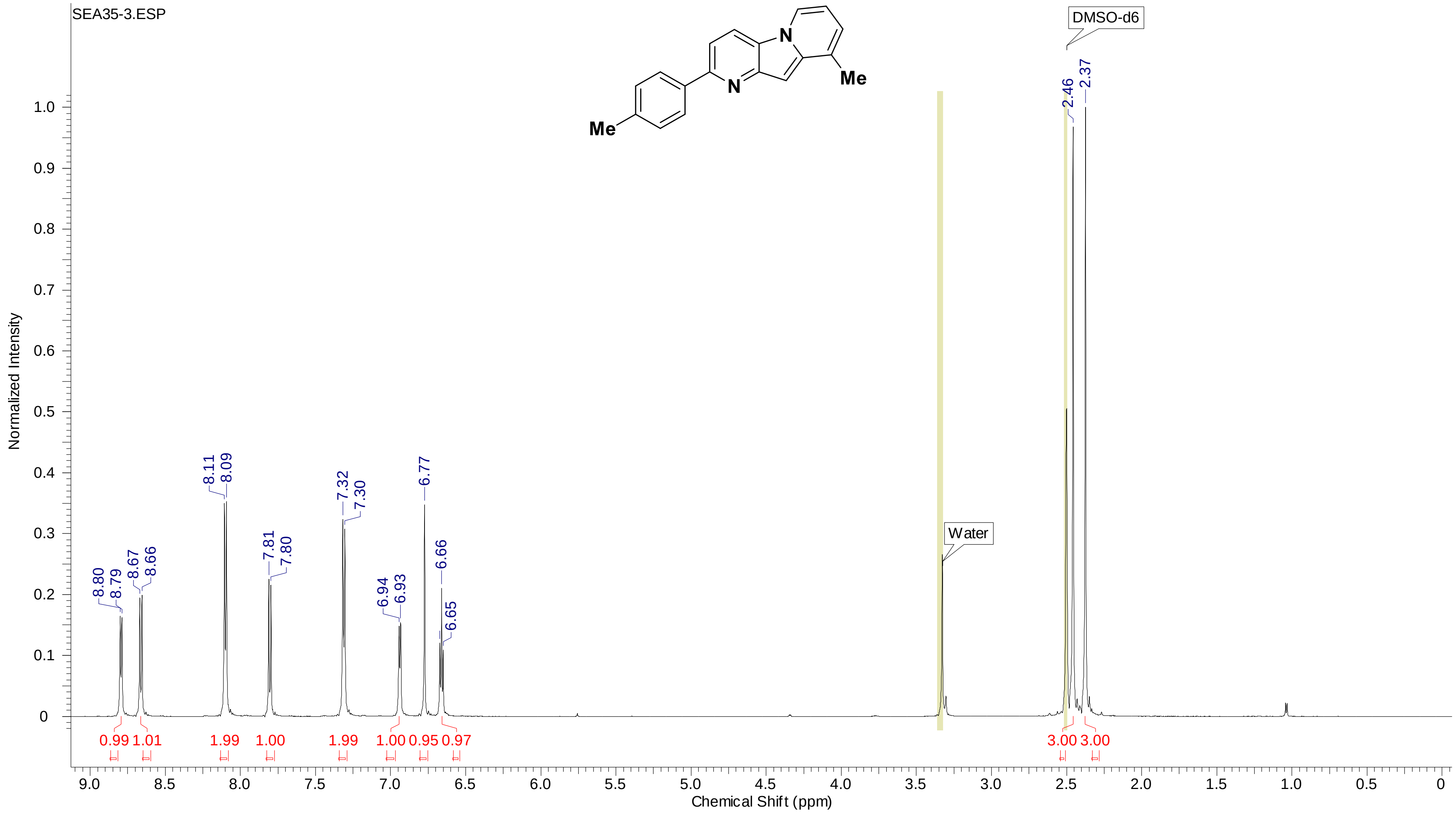

^{1}H NMR of compound **3e**

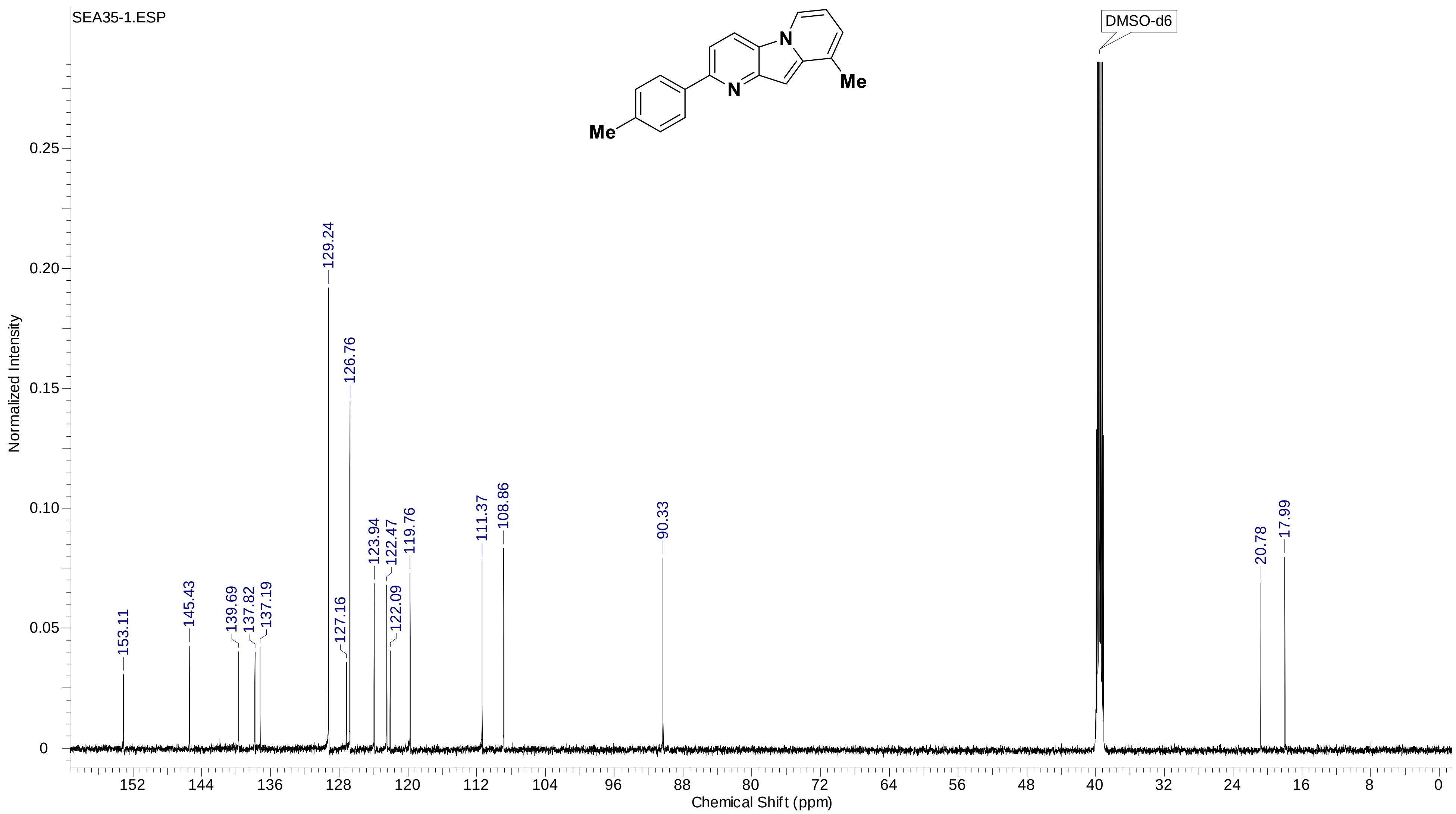

^{13}C NMR of compound **3e**

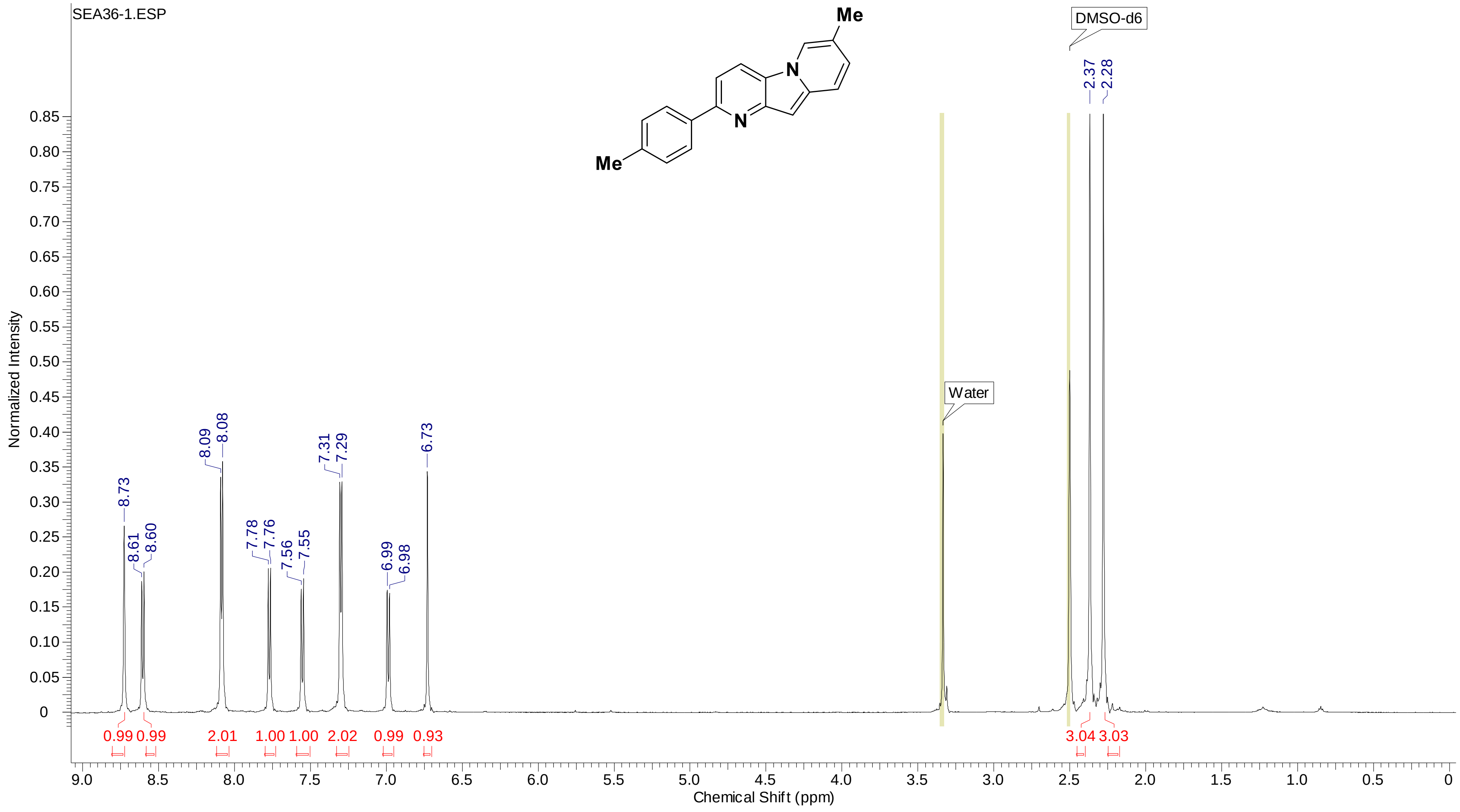

^{1}H NMR of compound **3f**

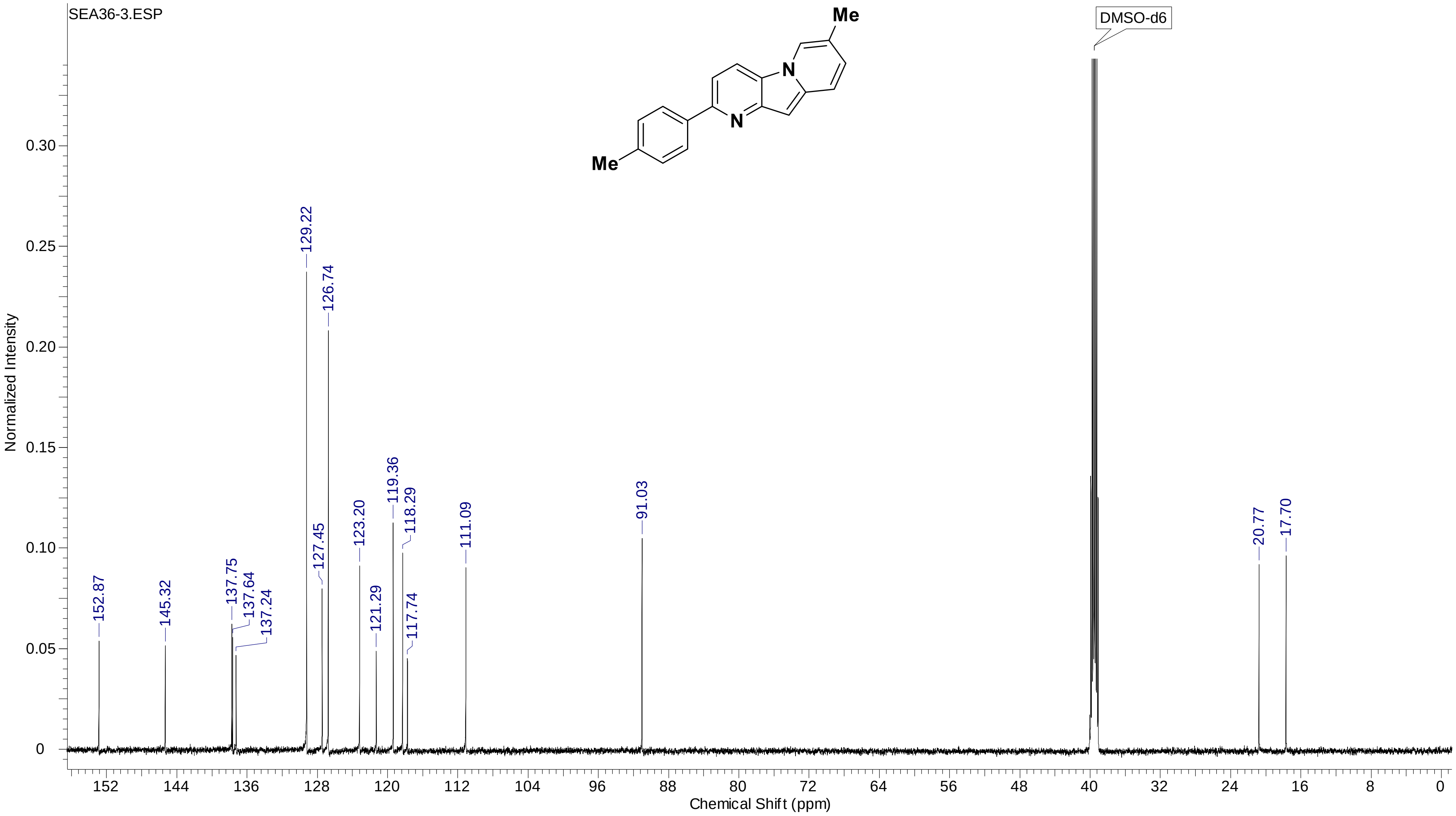

^{13}C NMR of compound **3f**

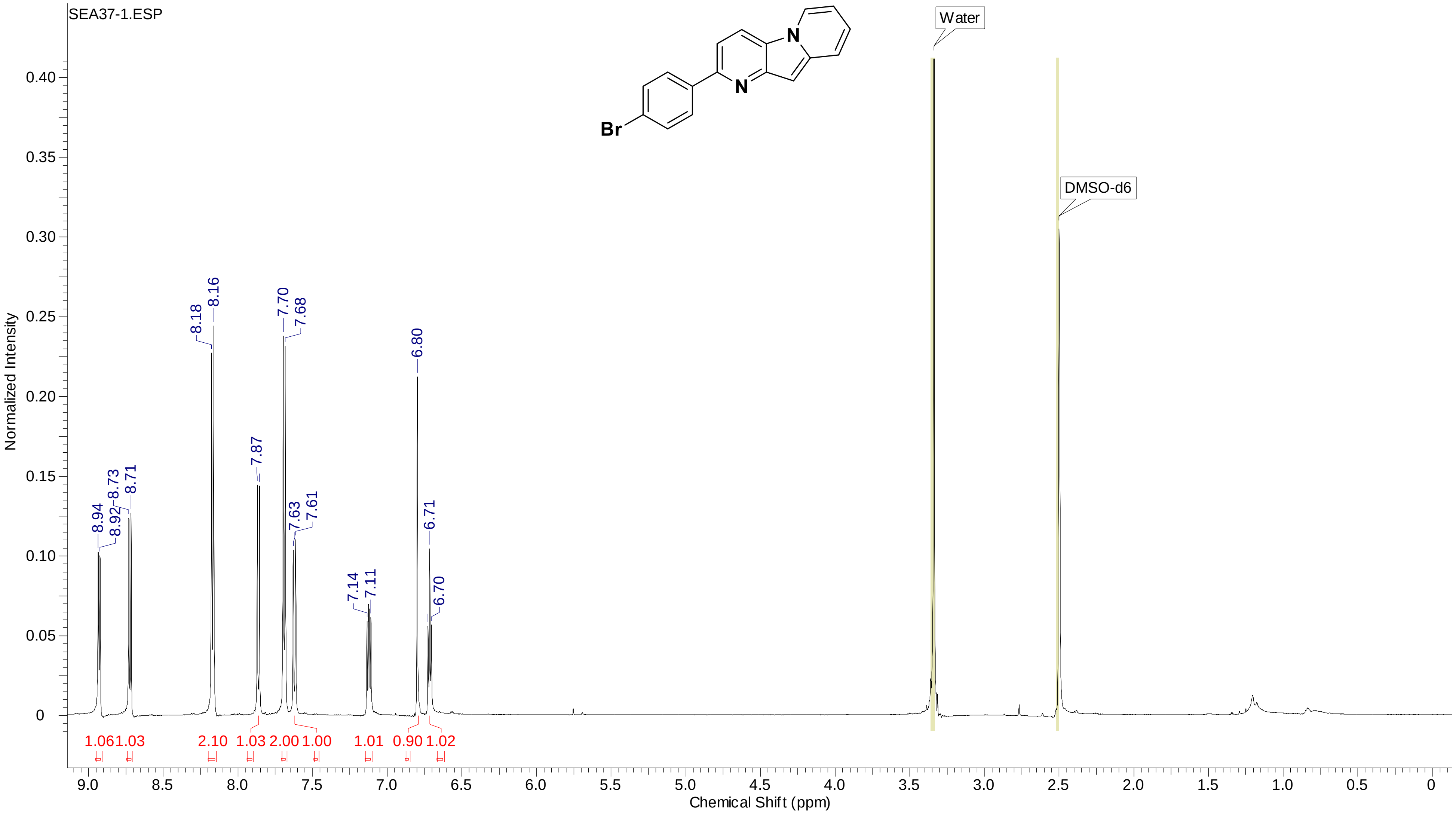

^{1}H NMR of compound **3g**

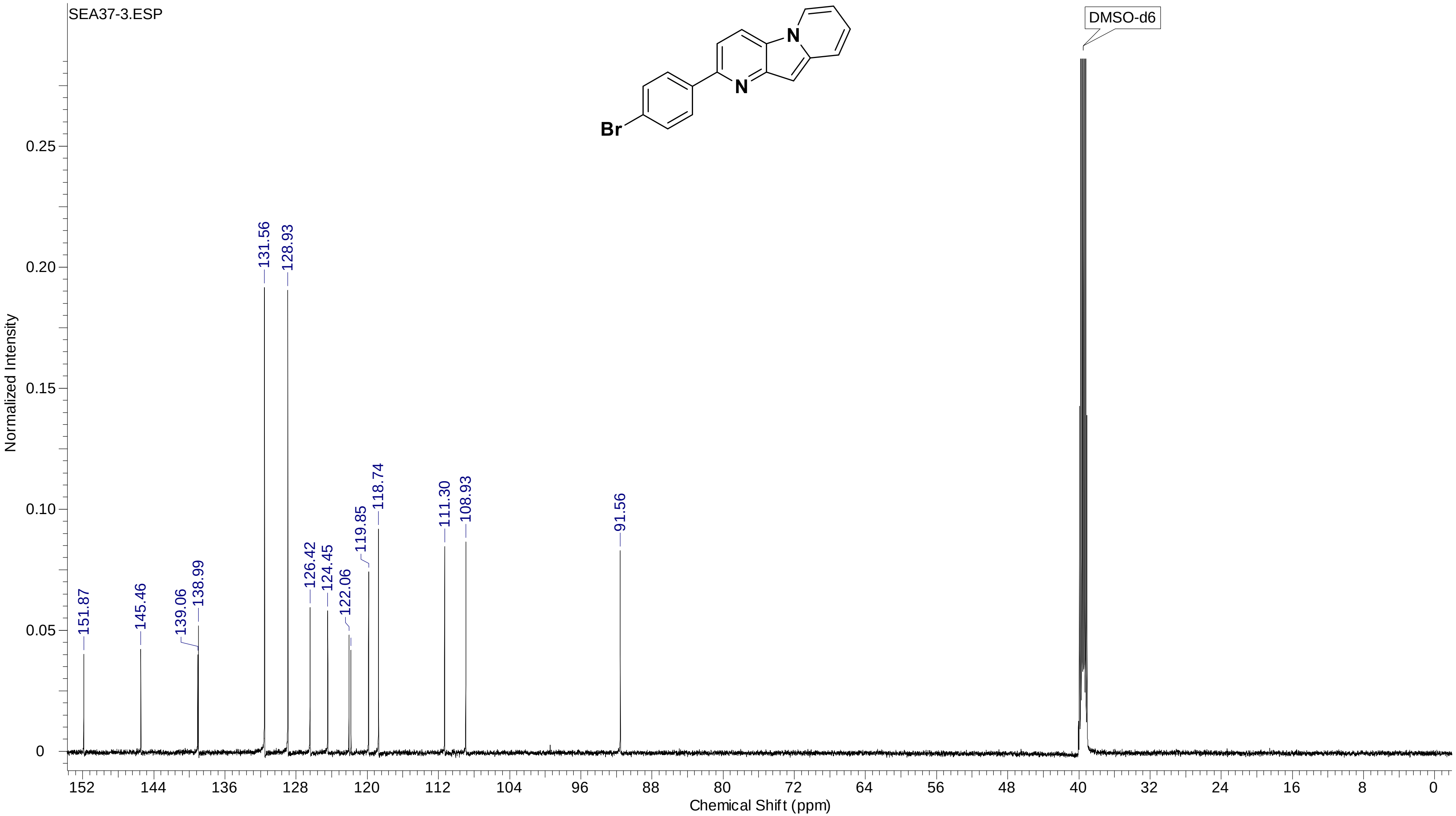

^{13}C NMR of compound **3g**

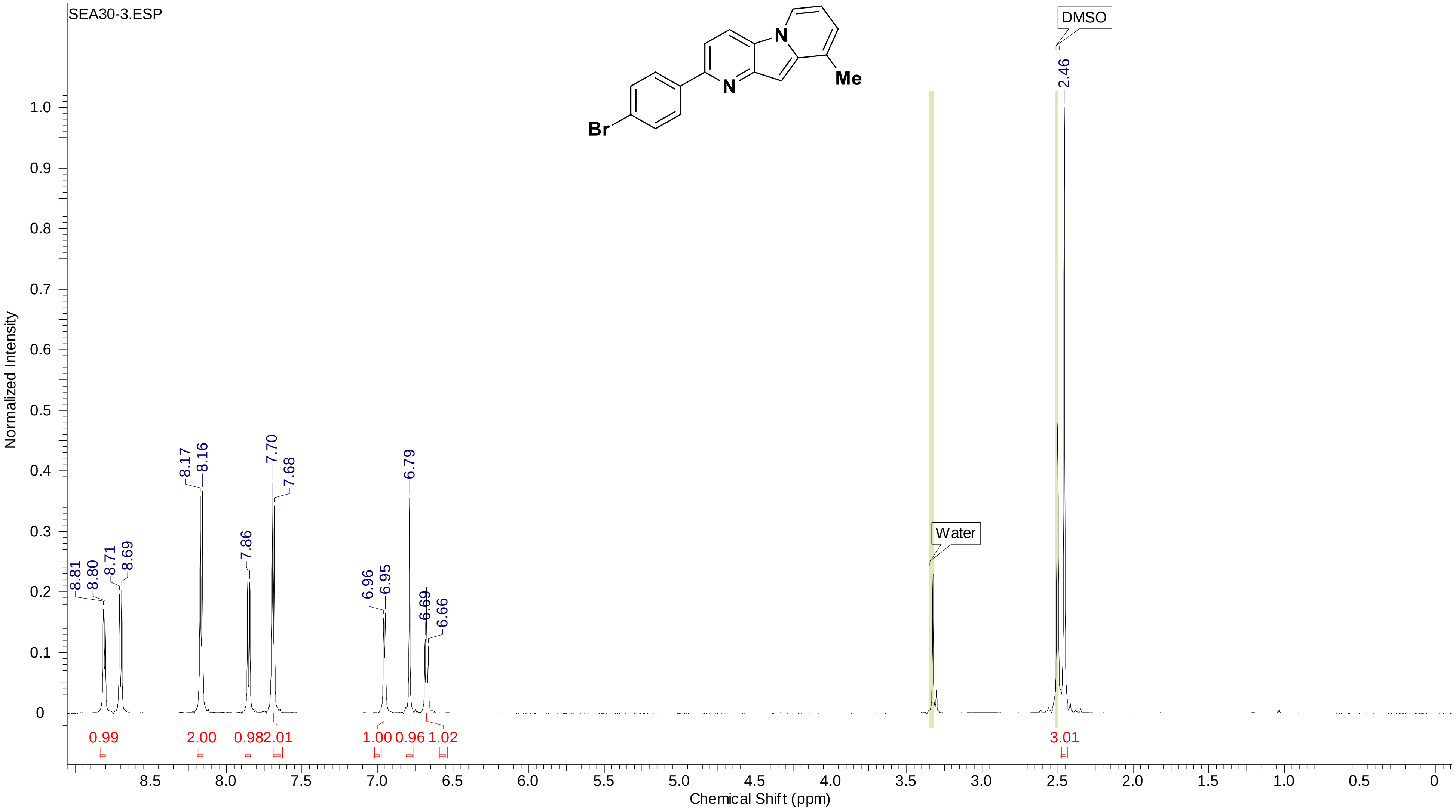

^{1}H NMR of compound **3h**

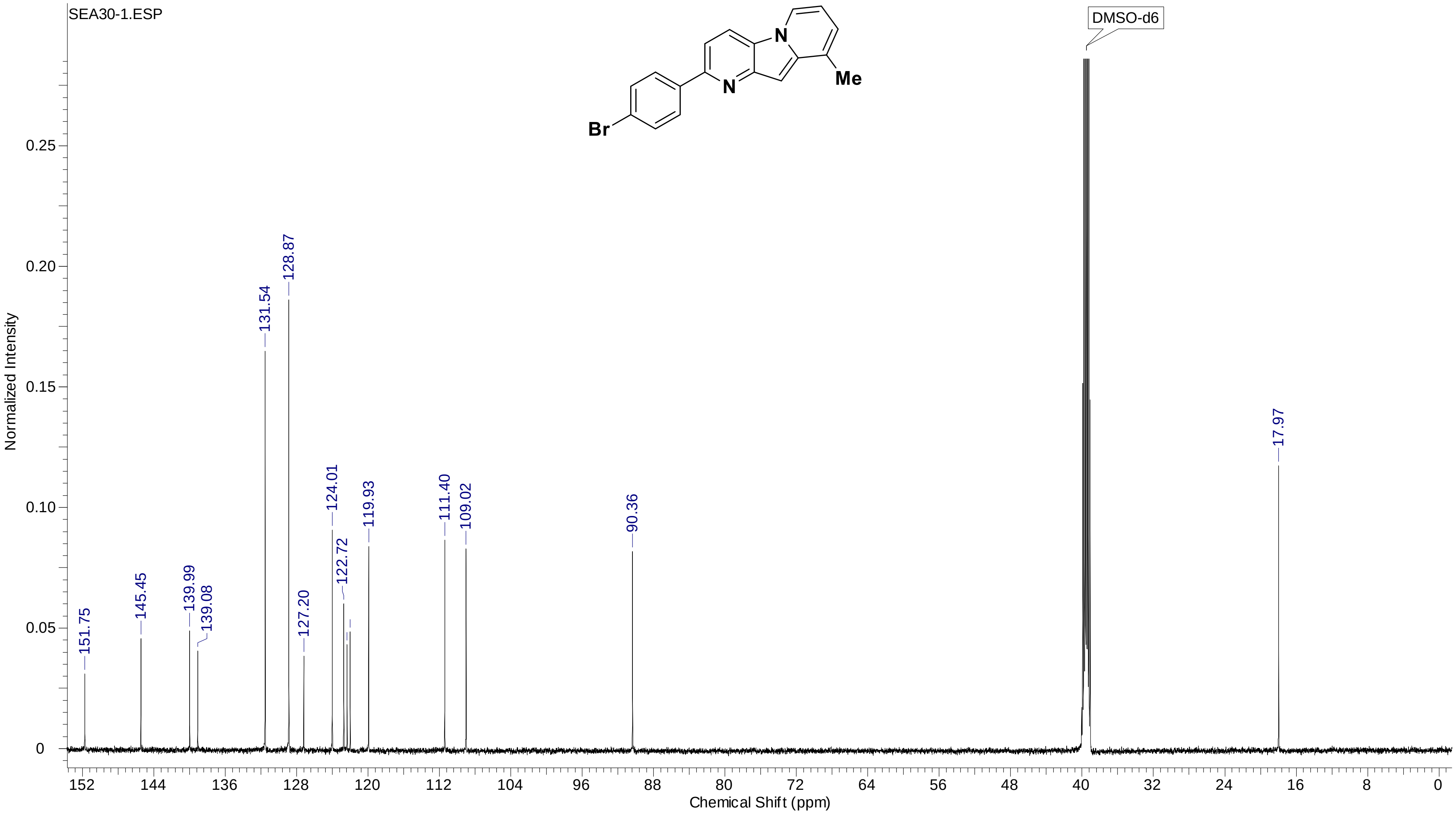

^{13}C NMR of compound **3h**

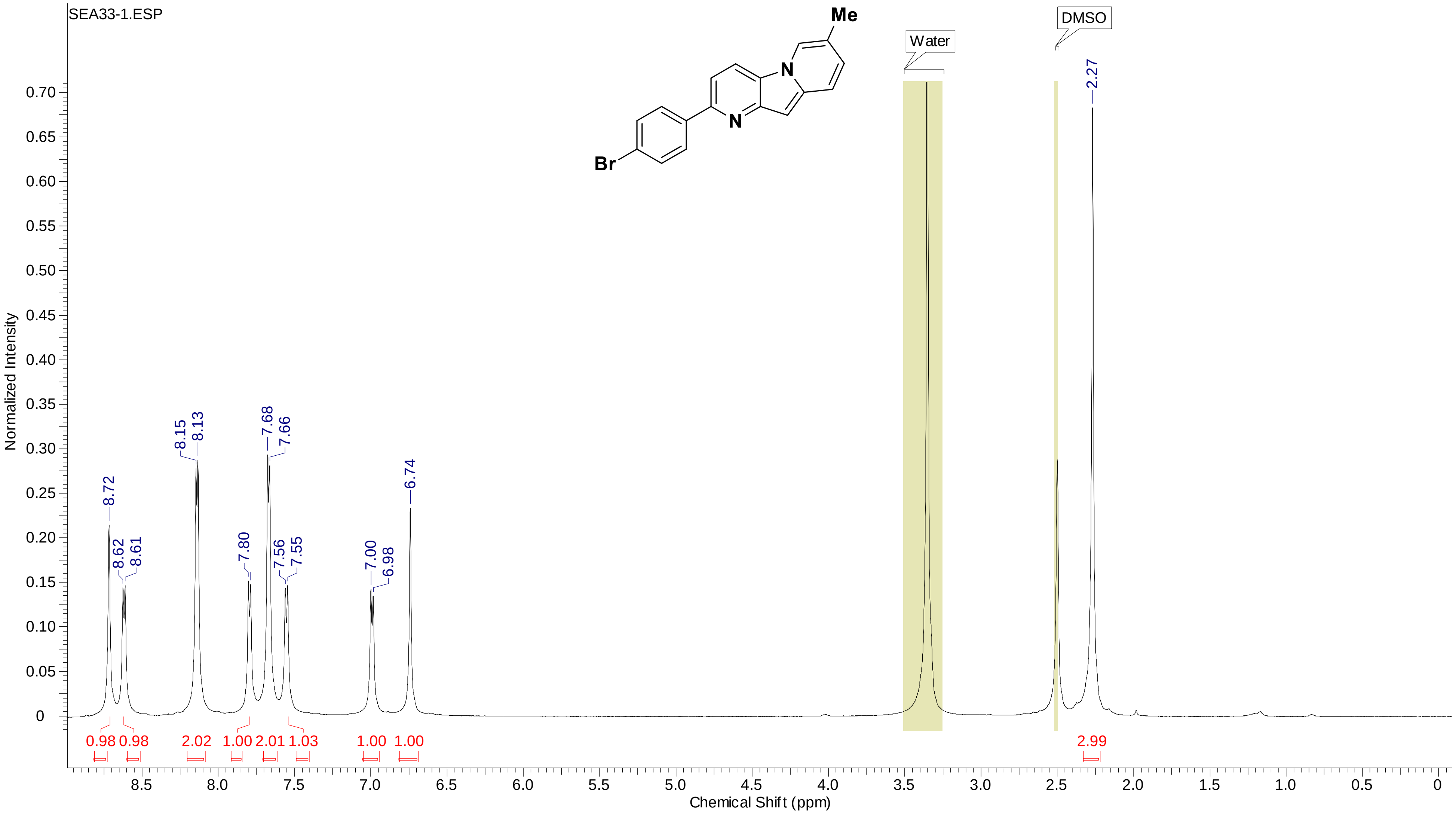

^{1}H NMR of compound **3i**

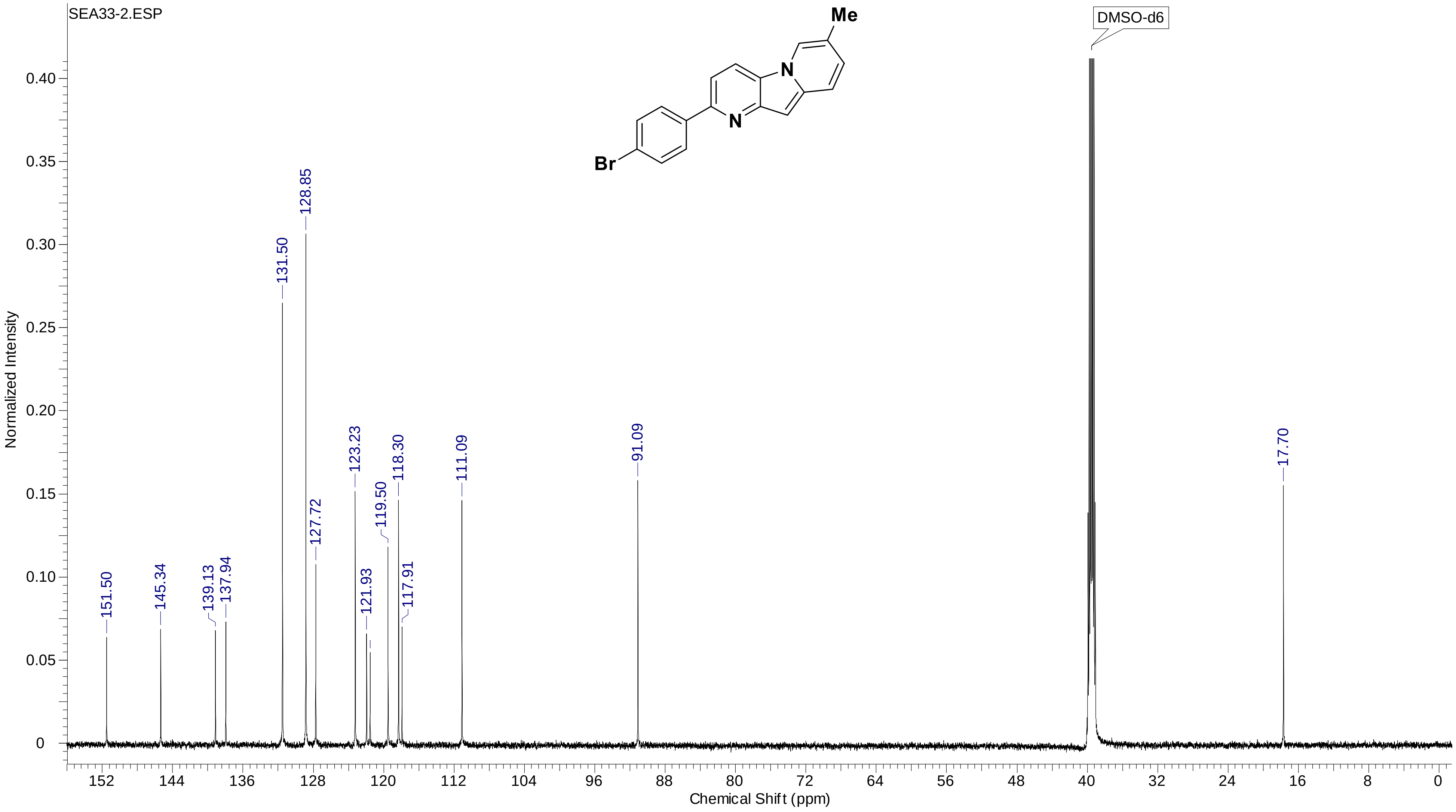

^{13}C NMR of compound **3i**

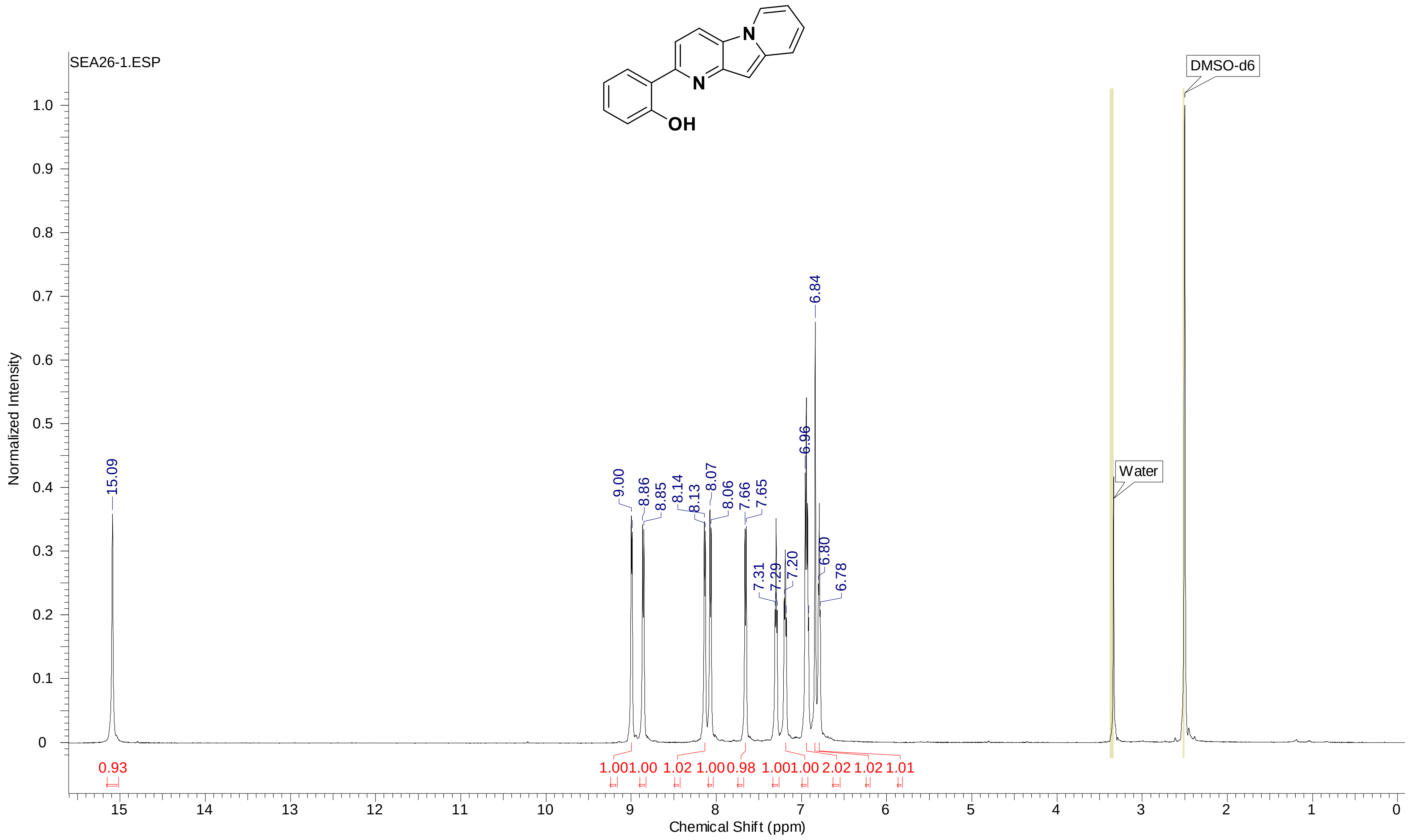

^{1}H NMR of compound **3j**

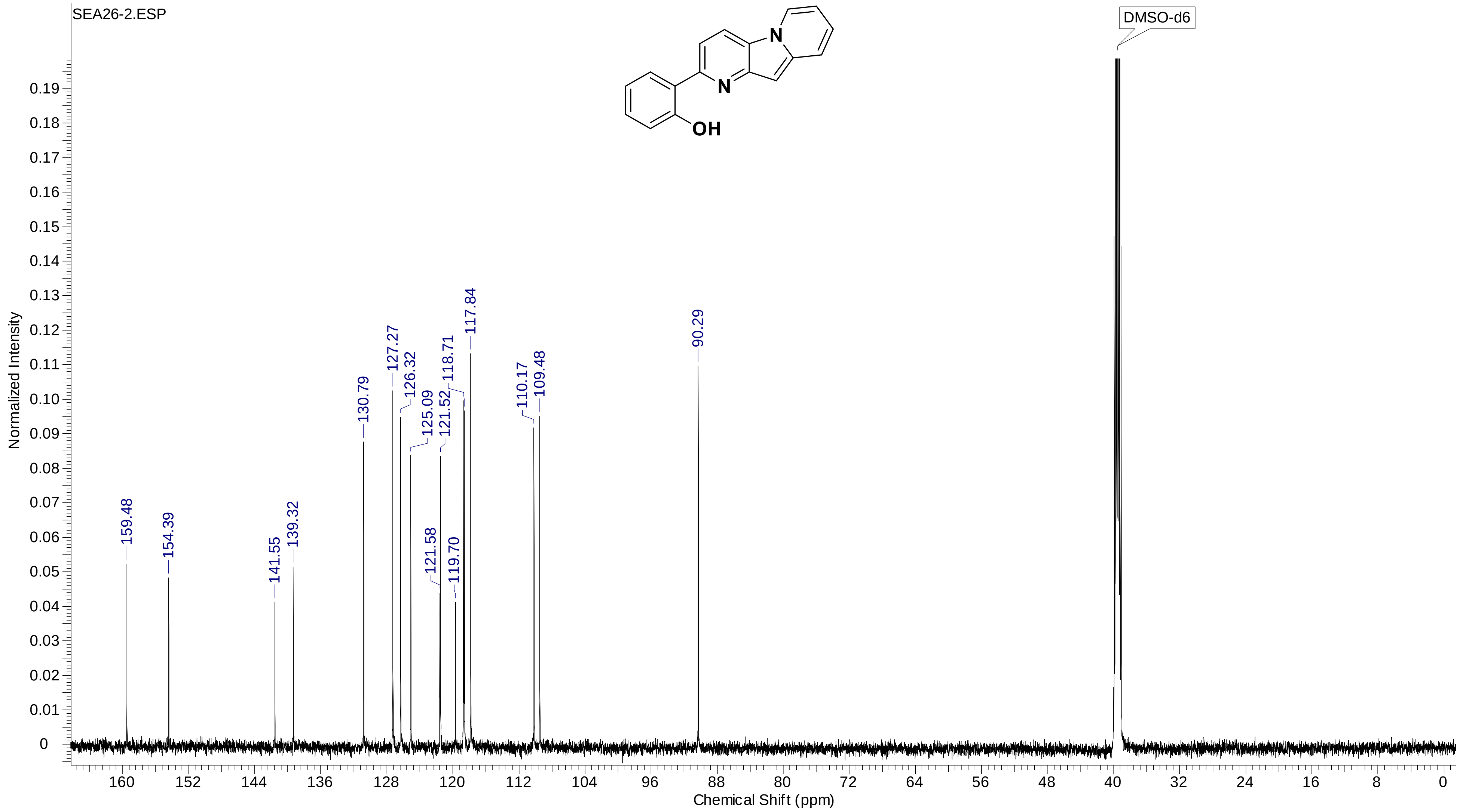

^{13}C NMR of compound **3j**

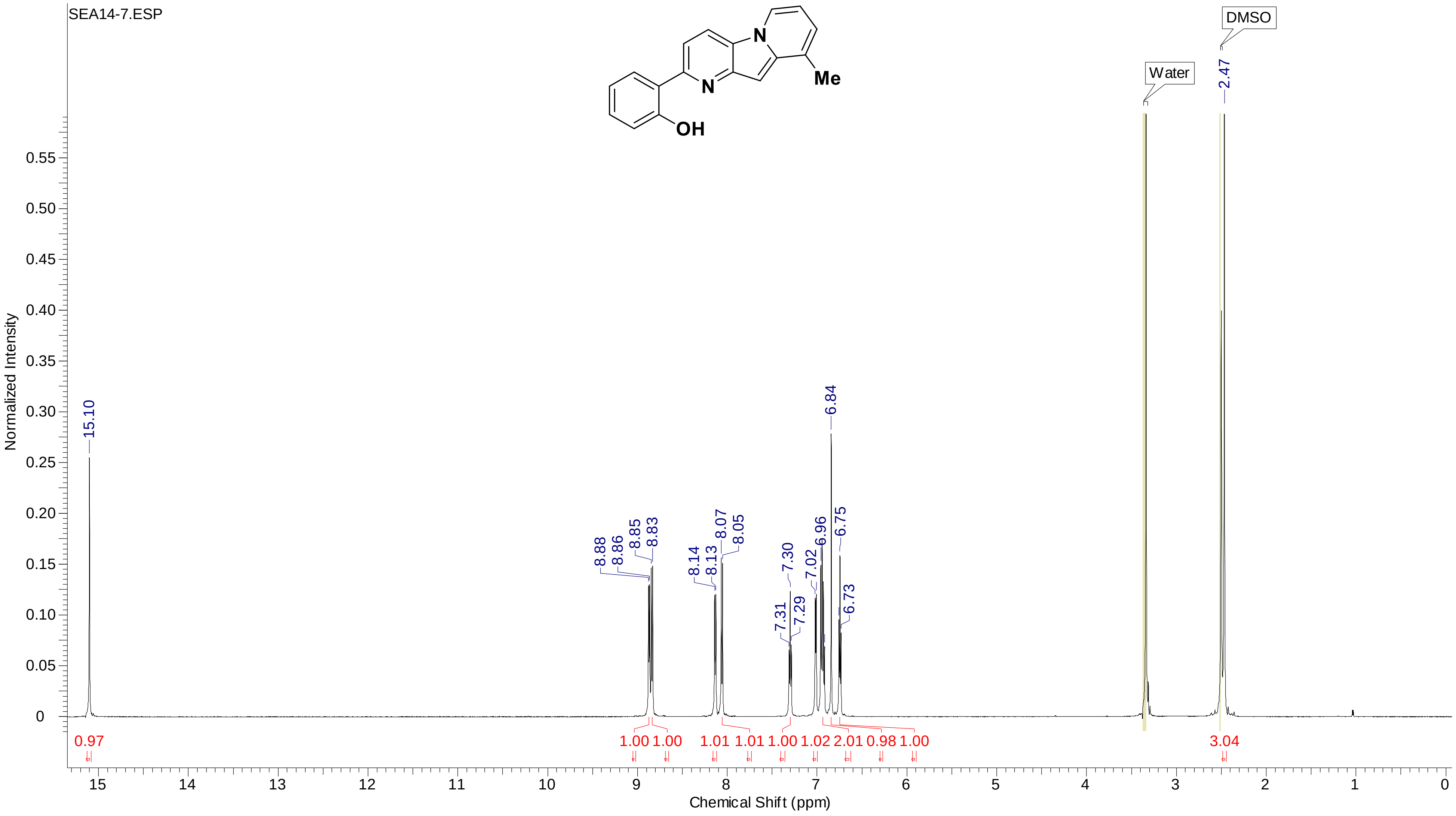

^{1}H NMR of compound **3k**

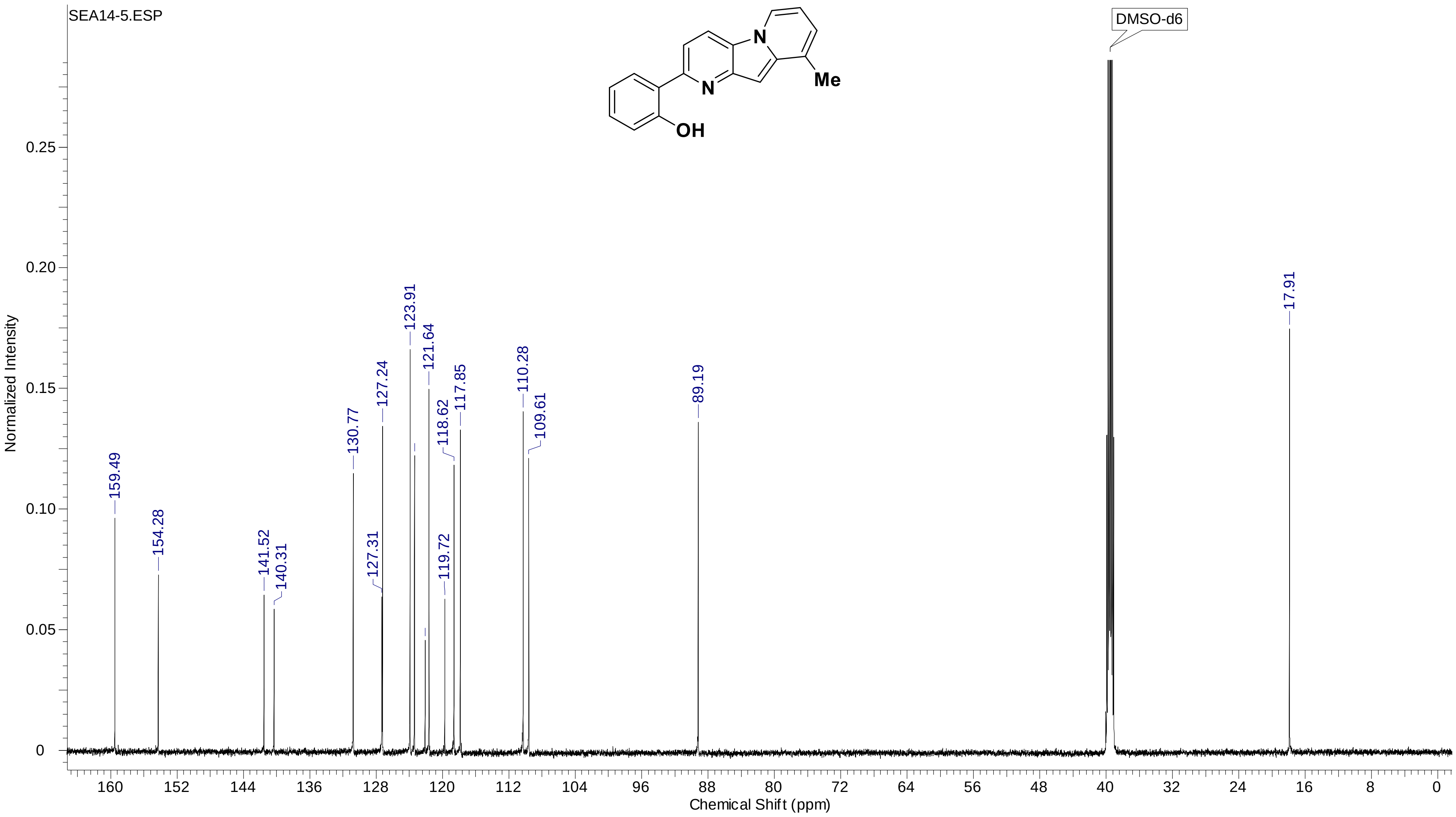

^{13}C NMR of compound **3k**

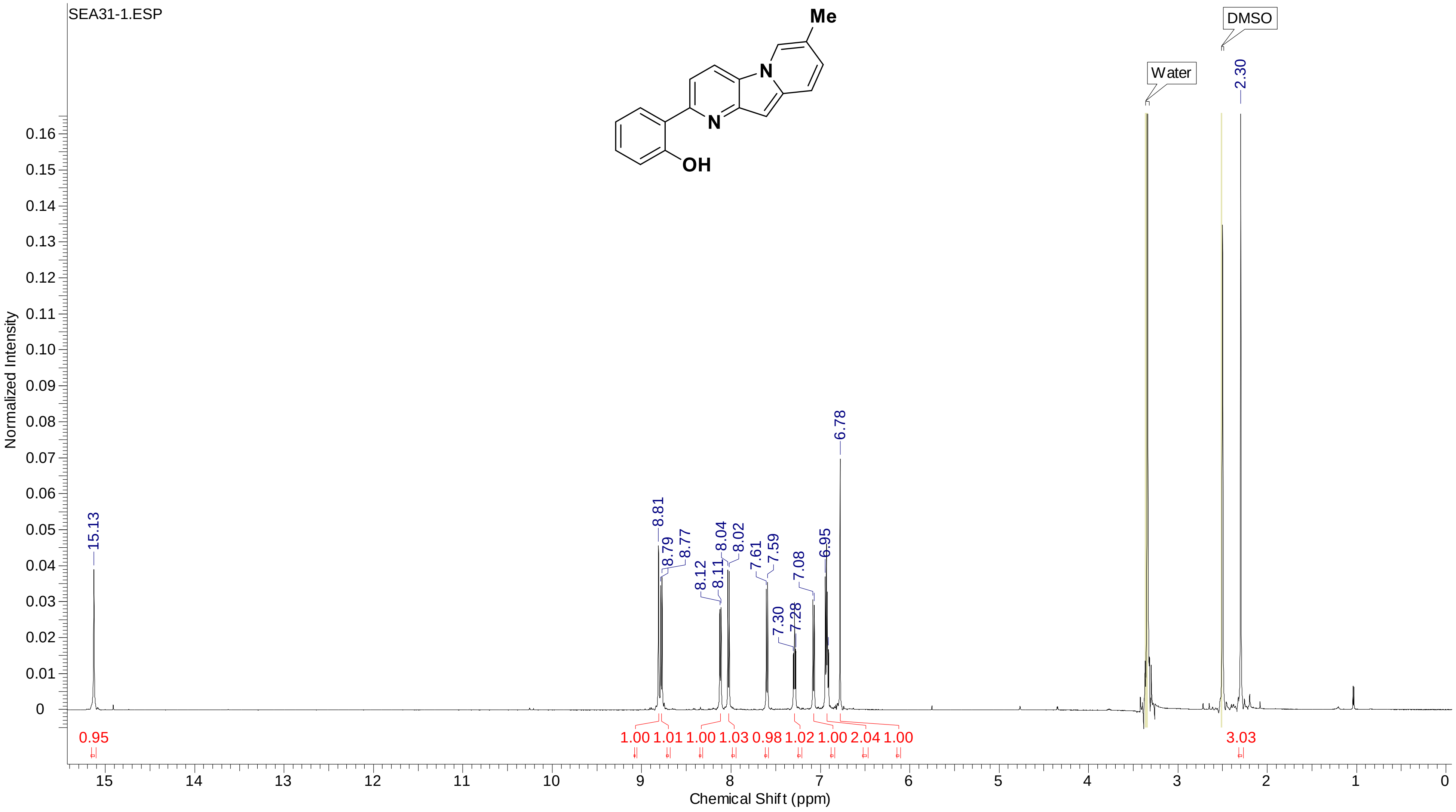

^{1}H NMR of compound **3l**

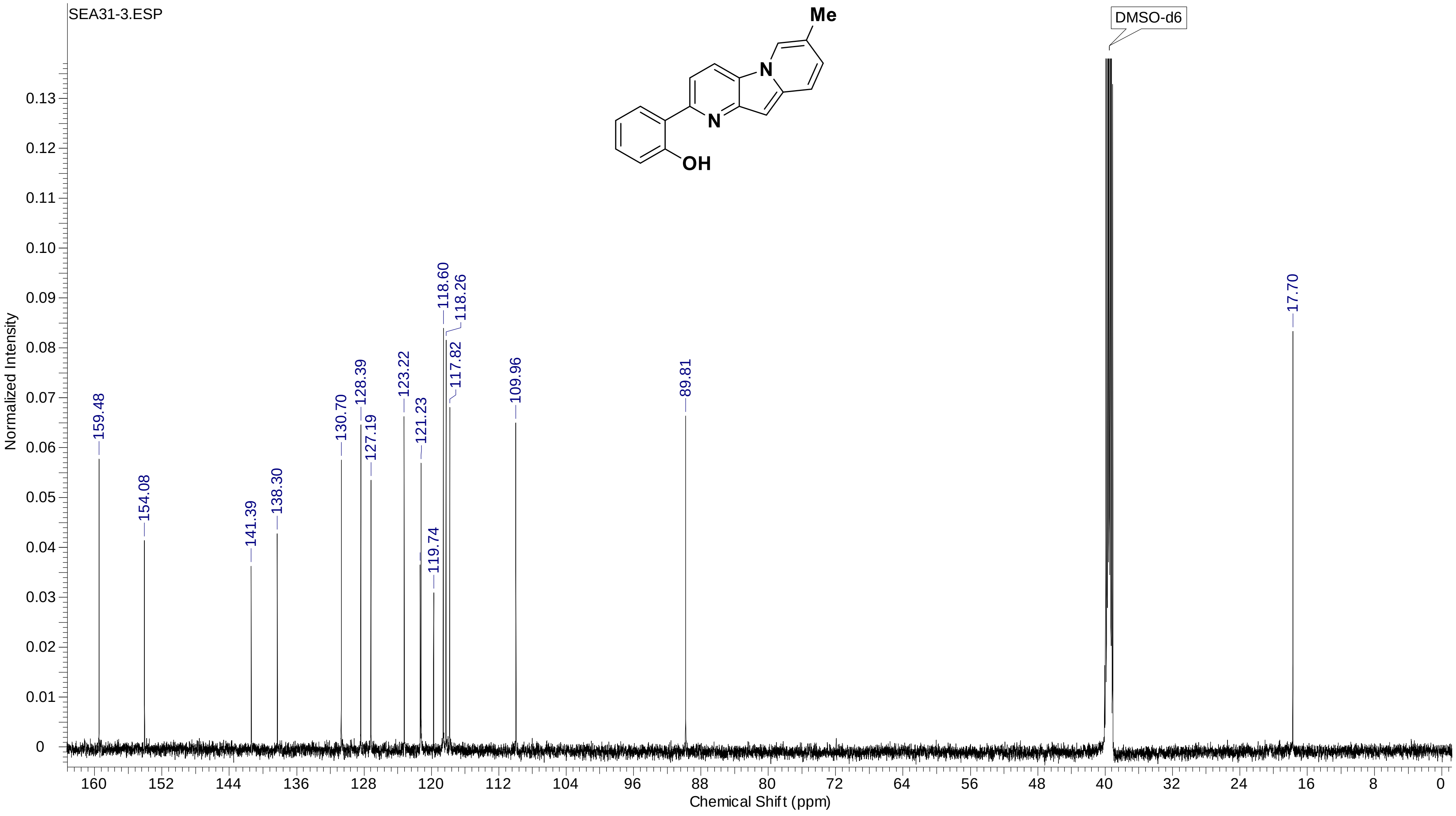

^{13}C NMR of compound **3l**

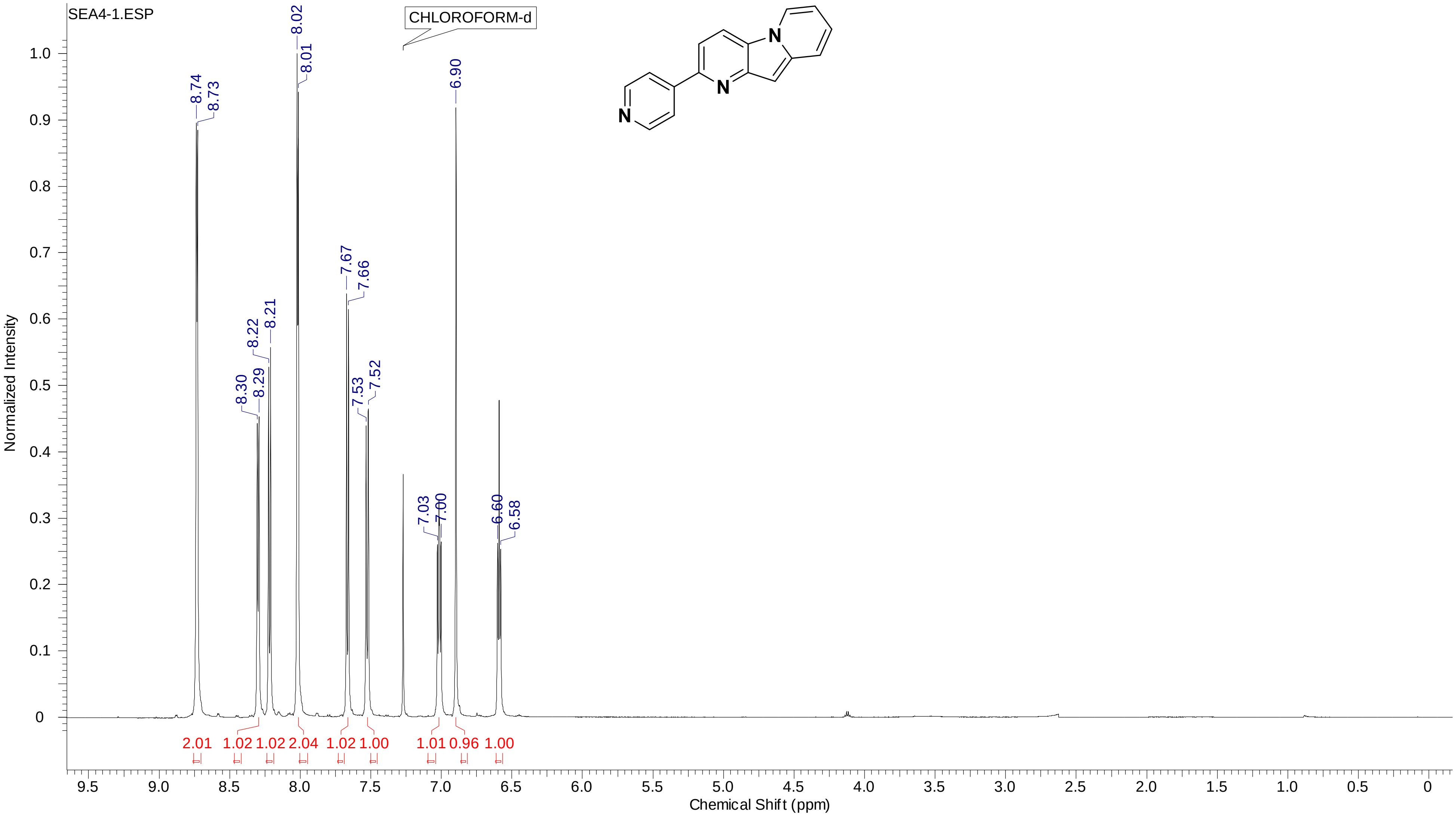

^{1}H NMR of compound **3m**

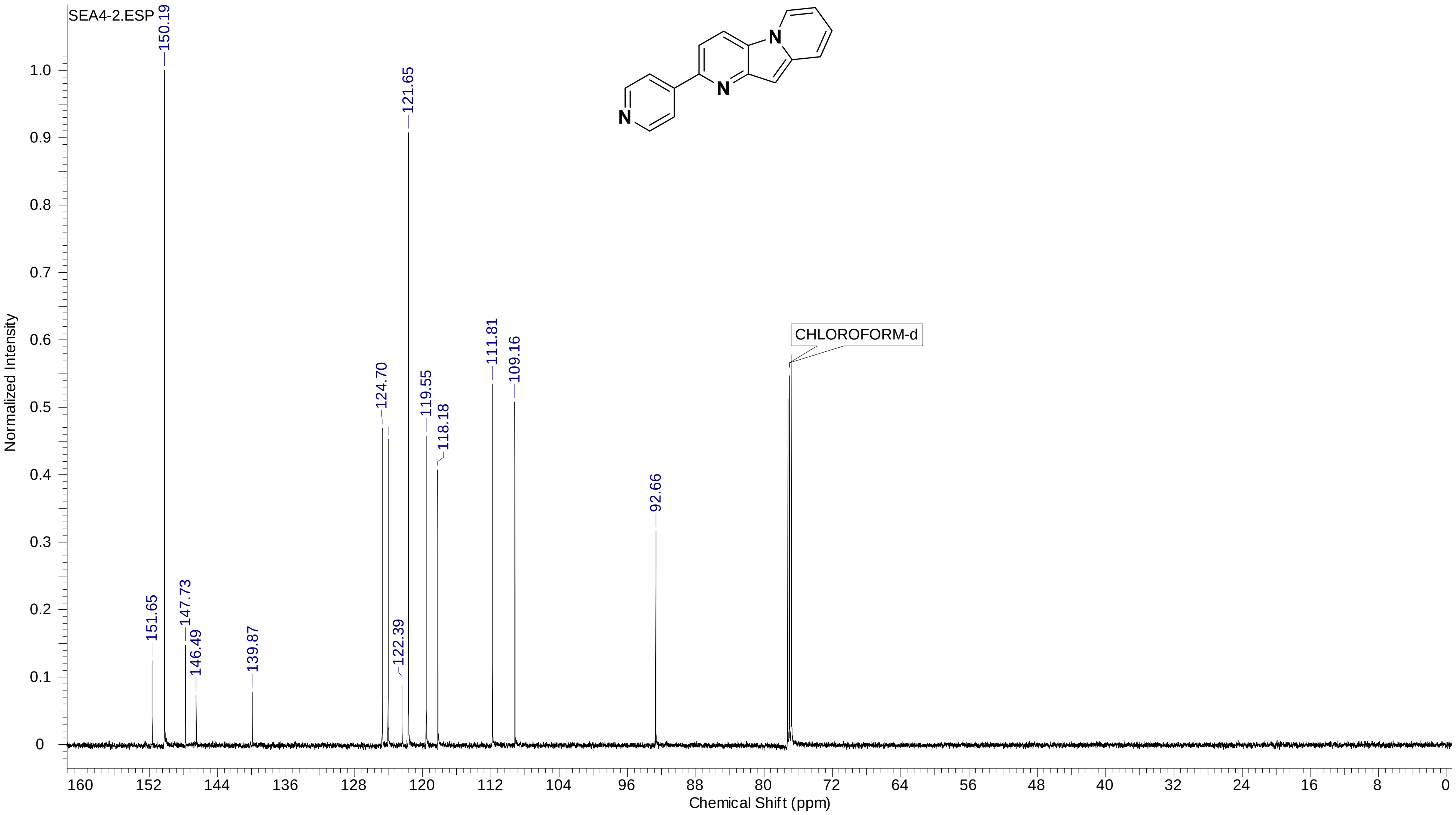

^{13}C NMR of compound **3m**

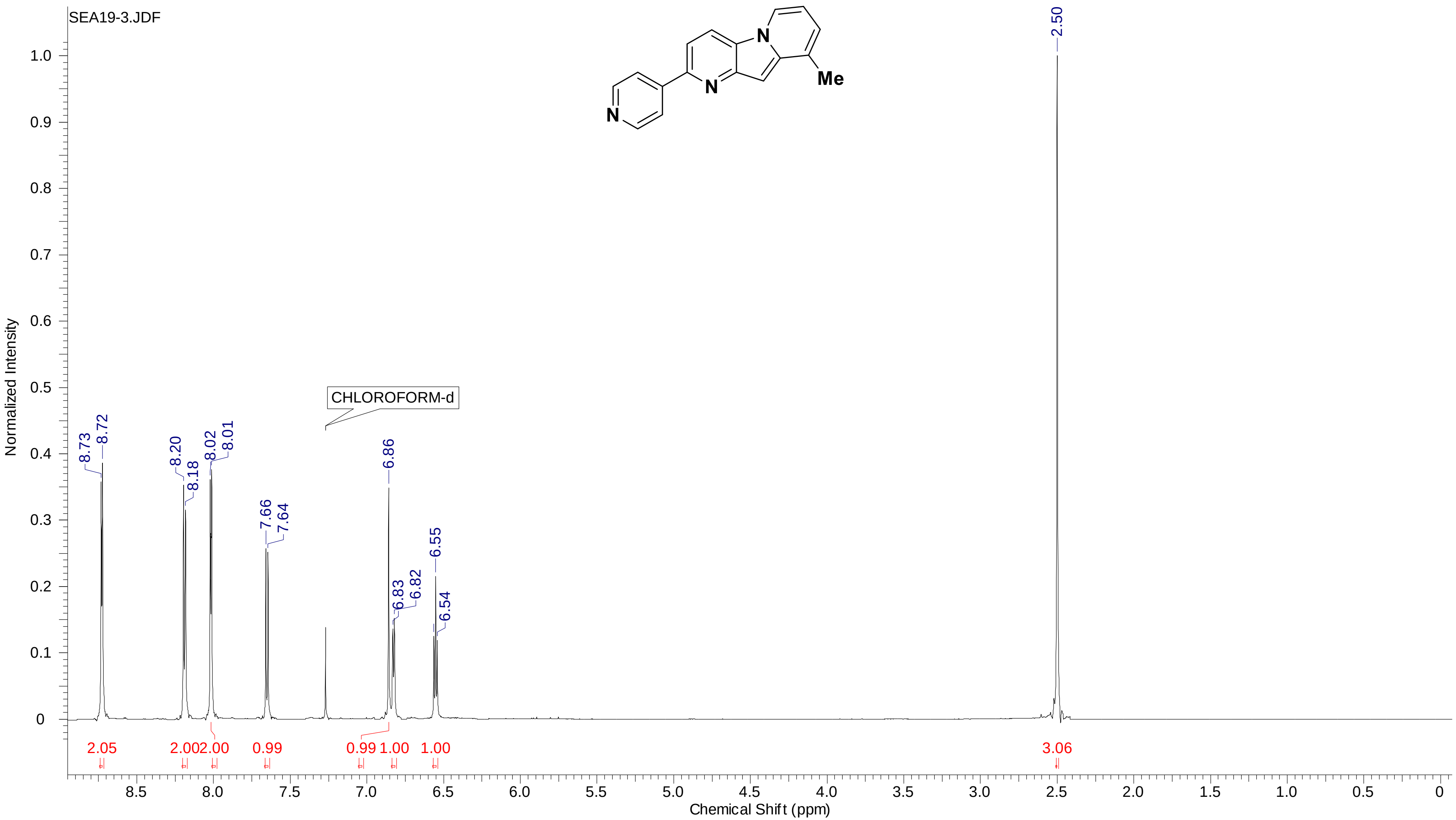

^{1}H NMR of compound **3n**

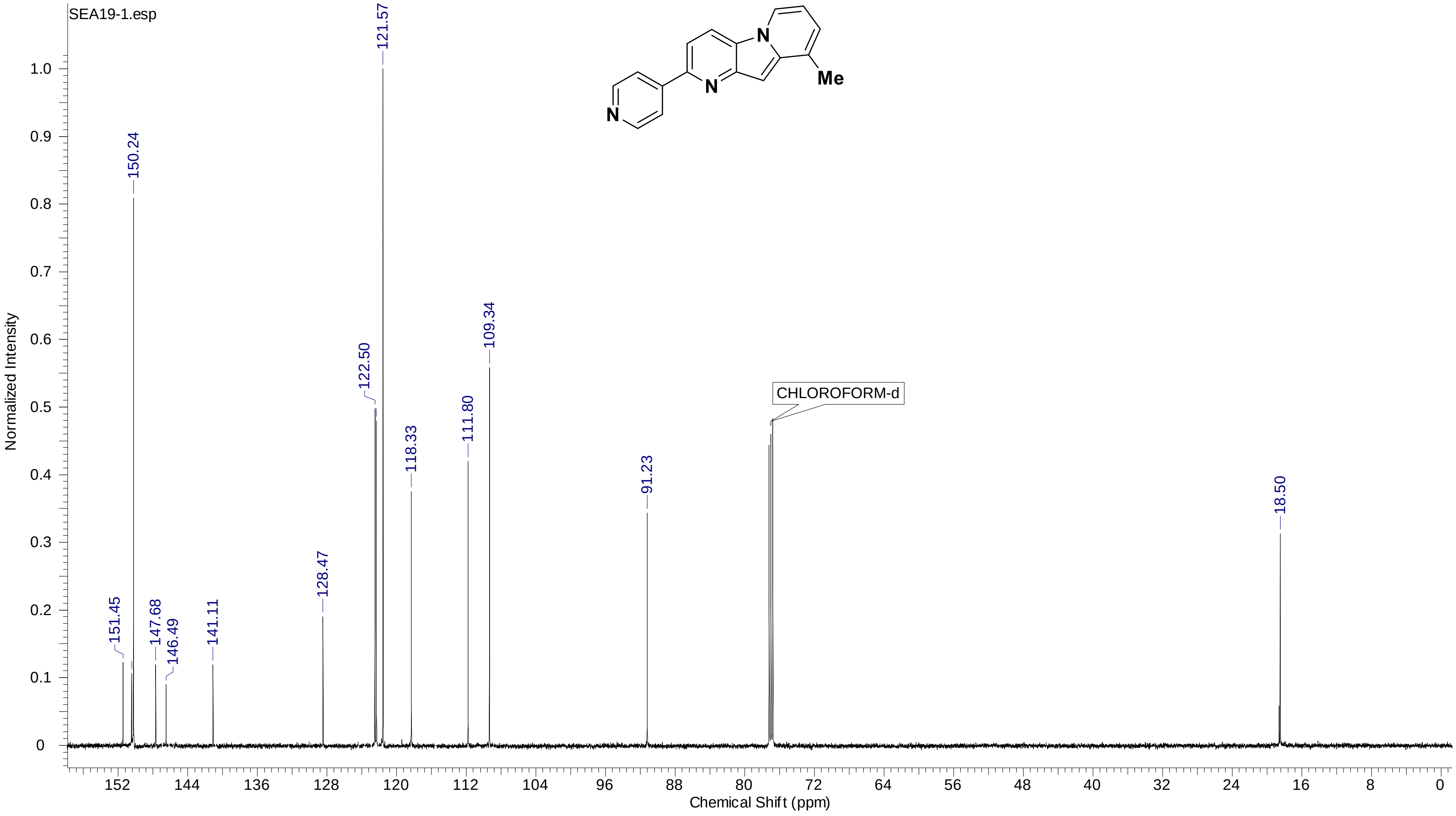

^{13}C NMR of compound **3n**

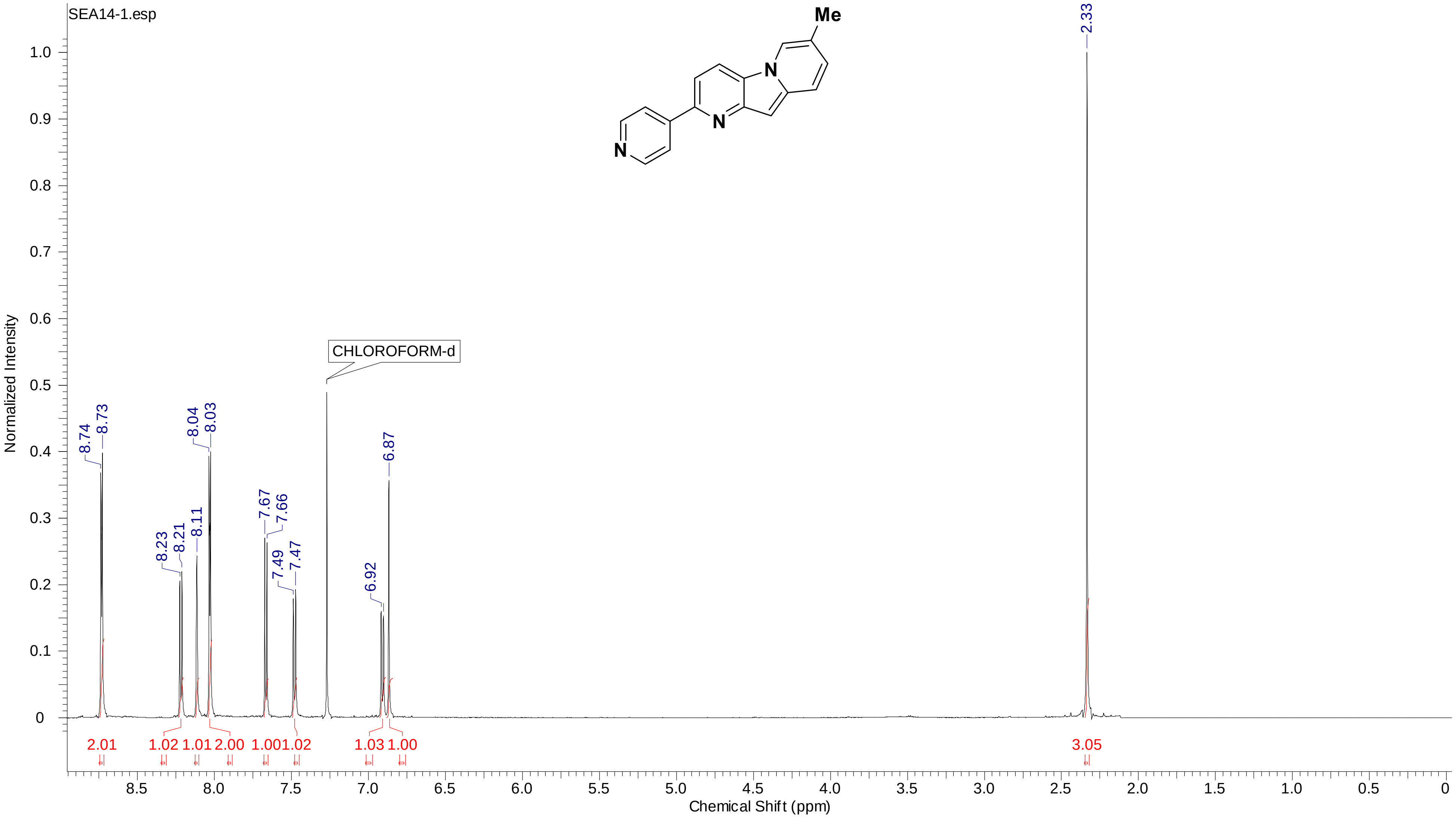

^{1}H NMR of compound **3o**

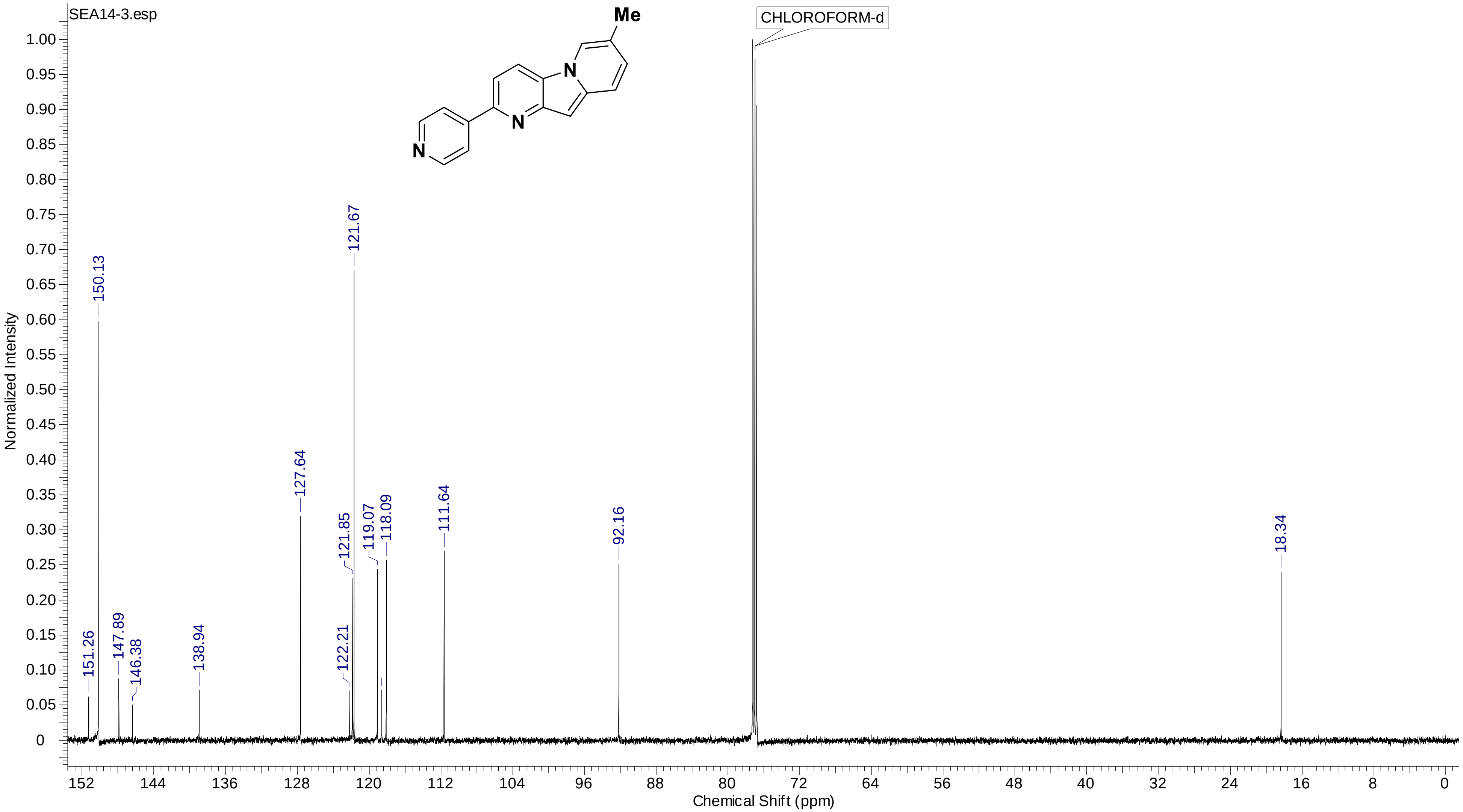

^{13}C NMR of compound **3o**

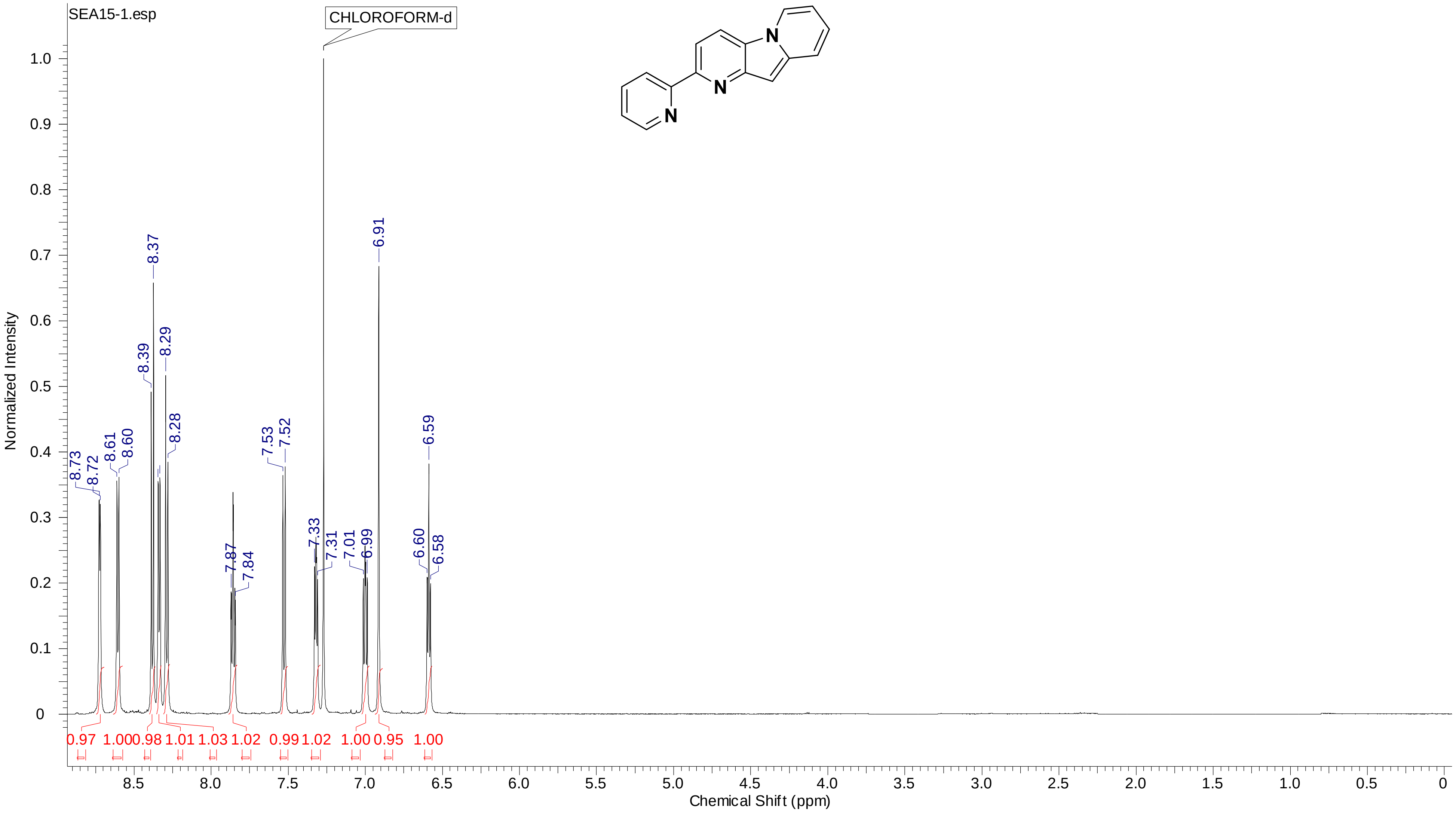

^{1}H NMR of compound **3p**

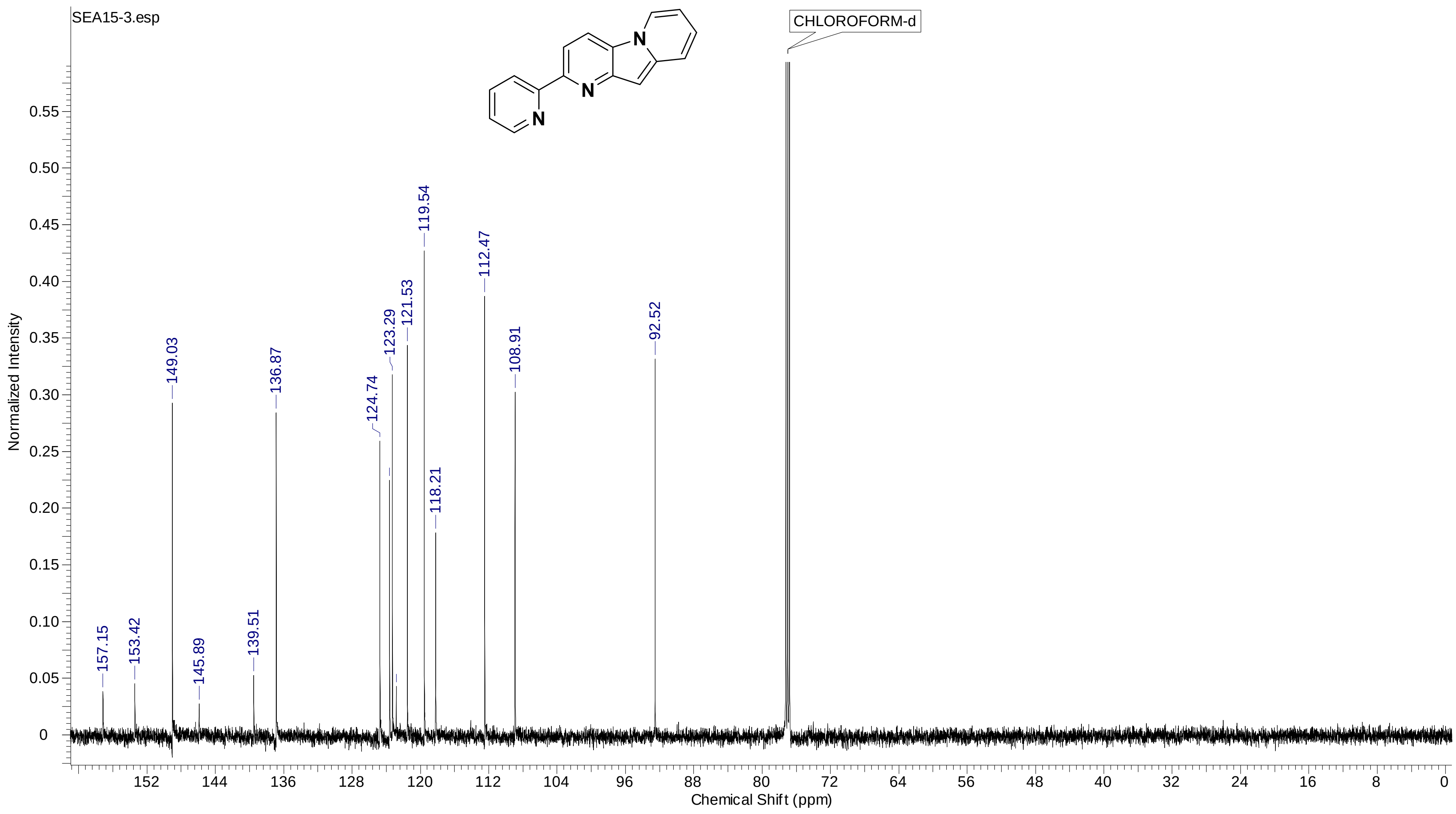

^{13}C NMR of compound **3p**

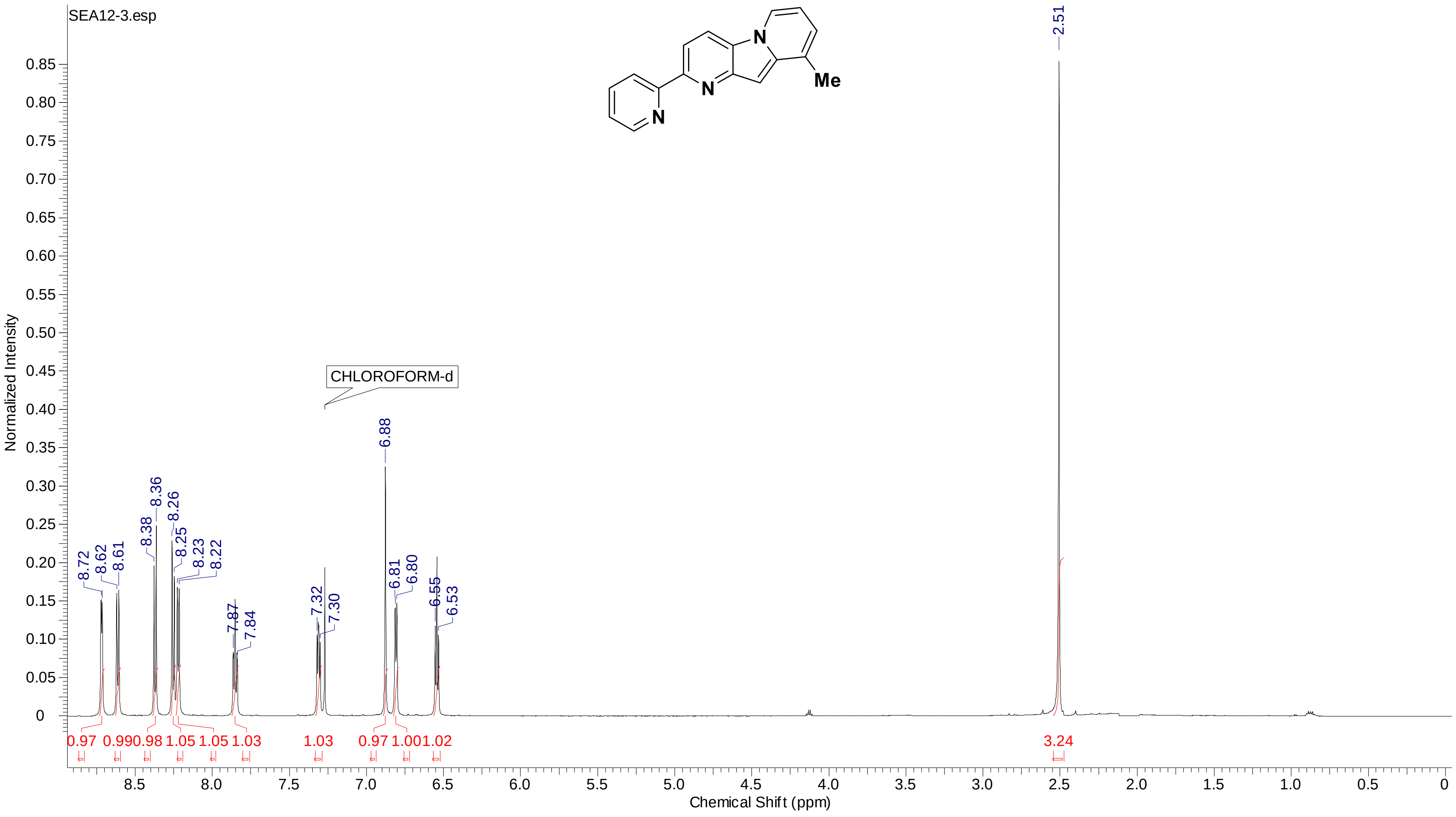

^{1}H NMR of compound **3q**

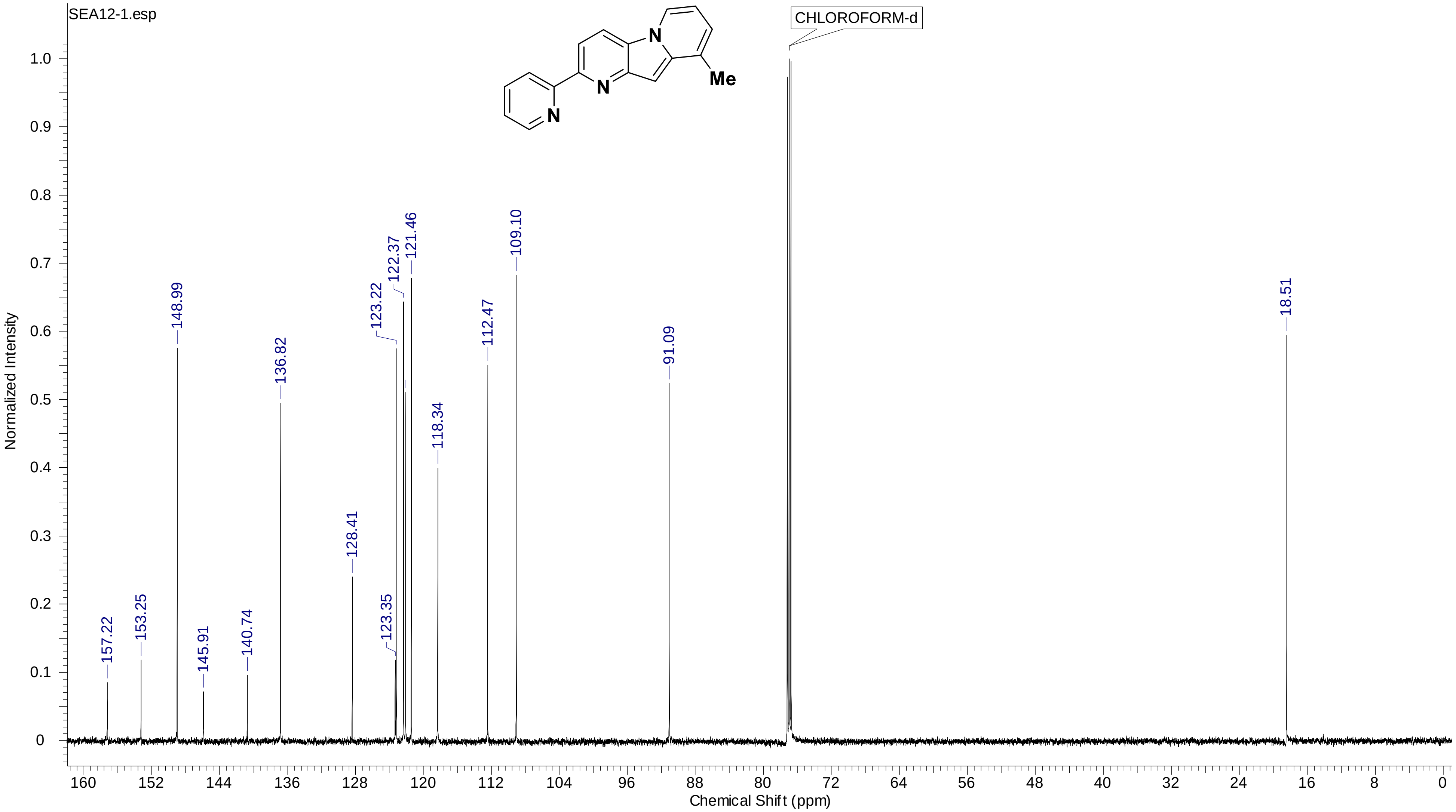

^{13}C NMR of compound **3q**

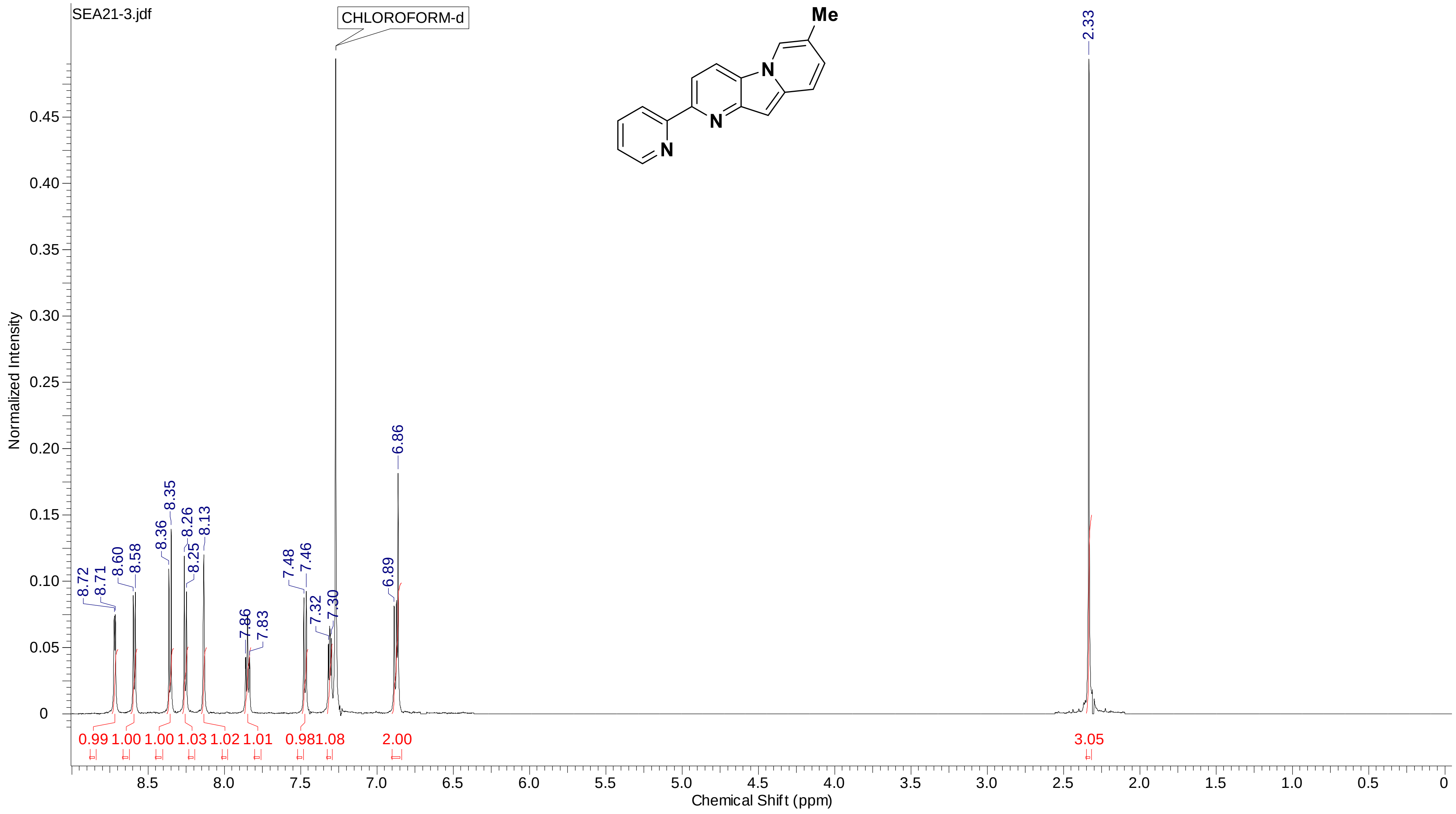

^{1}H NMR of compound **3r**

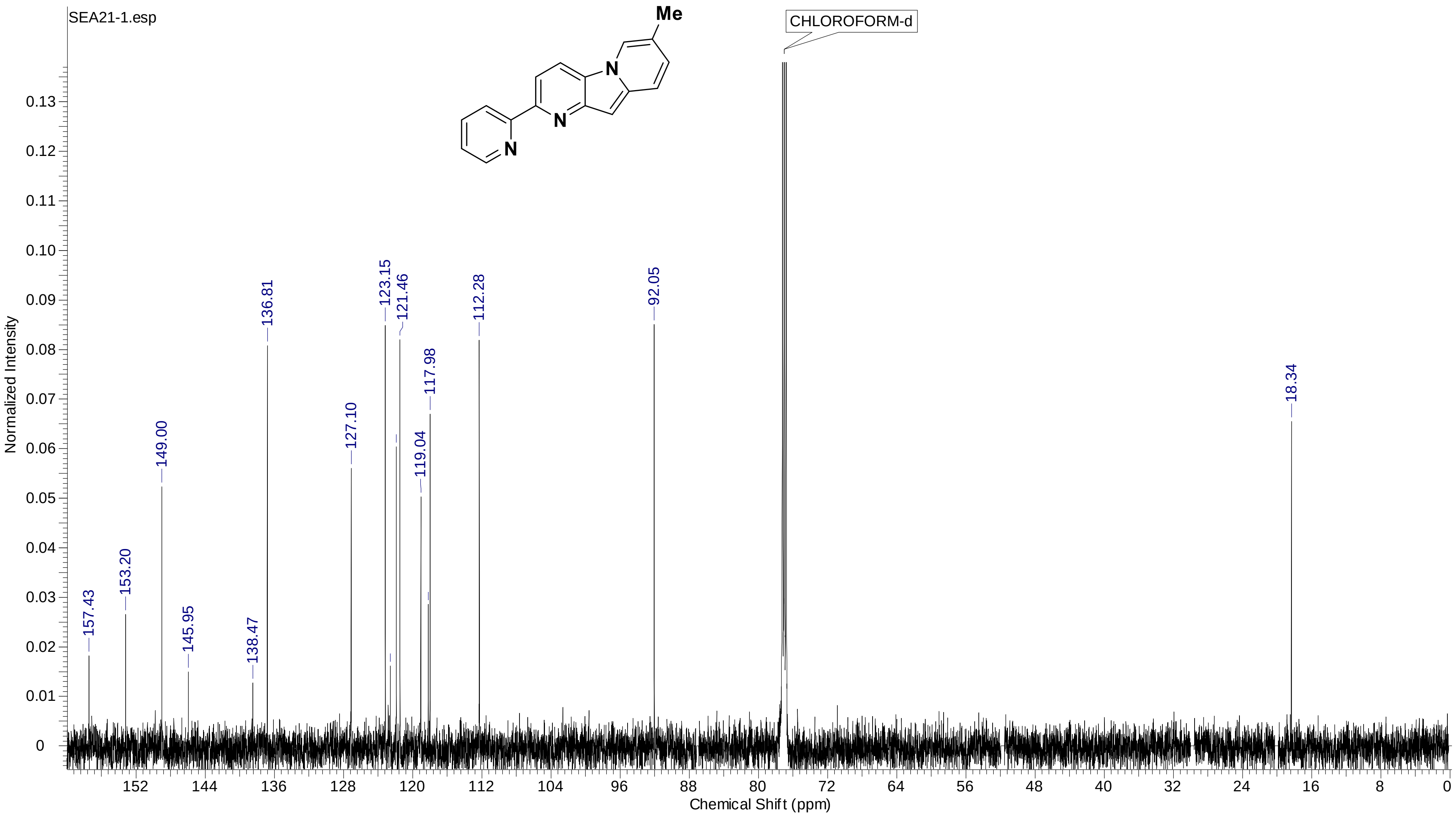

^{13}C NMR of compound **3r**

Absorption and fluorescence

The absorption spectrum was recorded at Varian Cary 100 UV/Vis Spectrophotometer at 190 nm – 1100 nm wavelength range; 0.1 mm optical path length cuvette is used for measurement. The fluorescence measurements were recorded between 400 nm – 800 nm wavelength range using Hitachi F-7100 spectrophotometer, samples were dissolved in toluene and 0.5 cm optical path length cuvette is used for measurement. Excitation wavelengths were taken at respective absorption spectral OD, to measure emission spectrum of each compound (Table 2) with the 150 W xenon lamp, self-deozonating lamp house (slit with for both excitation emission is 5.0 nm)

The fluorescence quantum yields of the samples were determined by the relative method using coumarin 153 (97% purity) in toluene as the reference (quantum yield = 0.82 [S1]) according to the following equation [S2]

$$\Phi_{f,x} = \Phi_{f,st} \times \frac{F_x}{F_{st}} \times \frac{A_{st}}{A_x} \times \frac{\eta_x^2}{\eta_{st}^2}$$

where x denotes sample and st – the reference; F is the integral photon flux in relative units for the spectrally- and blank-corrected spectra; A is the integral absorbance in the range of 400±10 nm, n is the refractive index of the solven

[S1] Robert Kro´licki , Włodzimierz Jarze¸ba, Mehran Mostafavi, Isabelle Lampre, *J. Phys. Chem. A* **2002**, *106,* 1708-1713.

[S2] C. Würth, M. Grabolle, J. Pauli, M. Spieles, U. Resch-Genger, *Nat. Protoc.* **2013**, 8, 1535–1550.

Absorption and fluorescence spectra for studied compounds in toluene

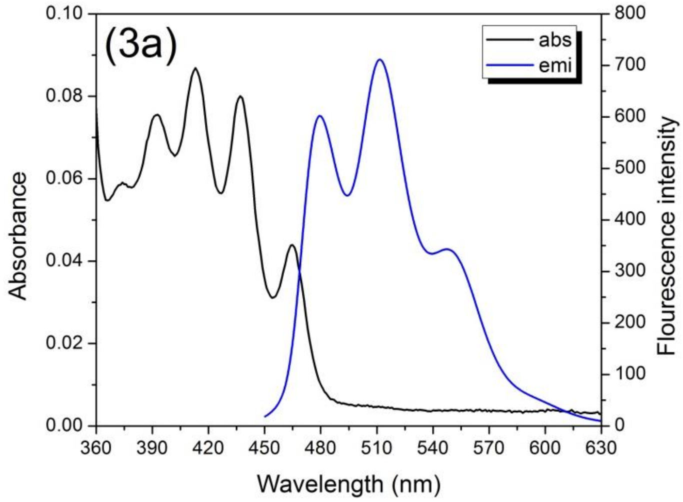

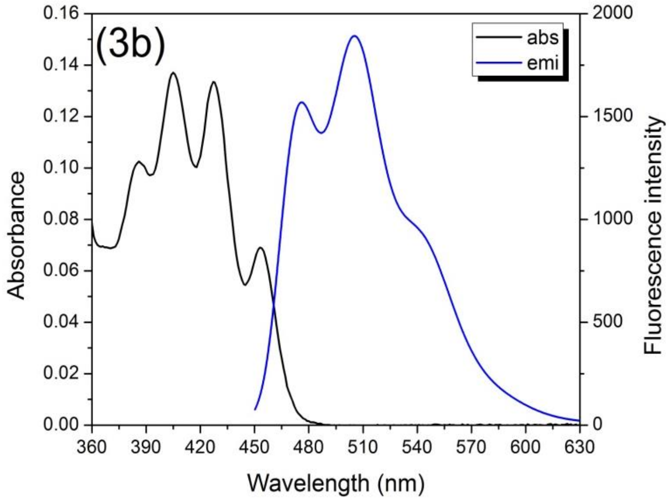

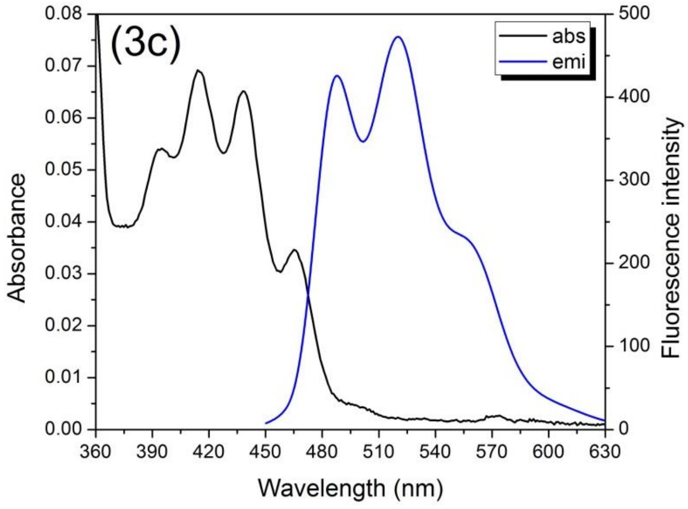

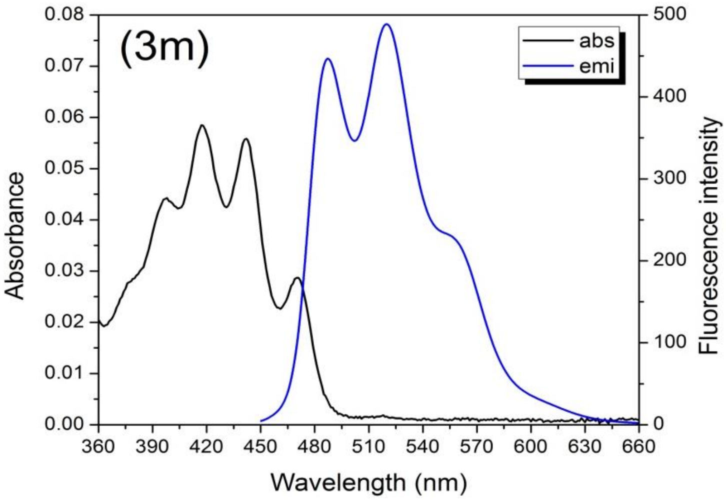

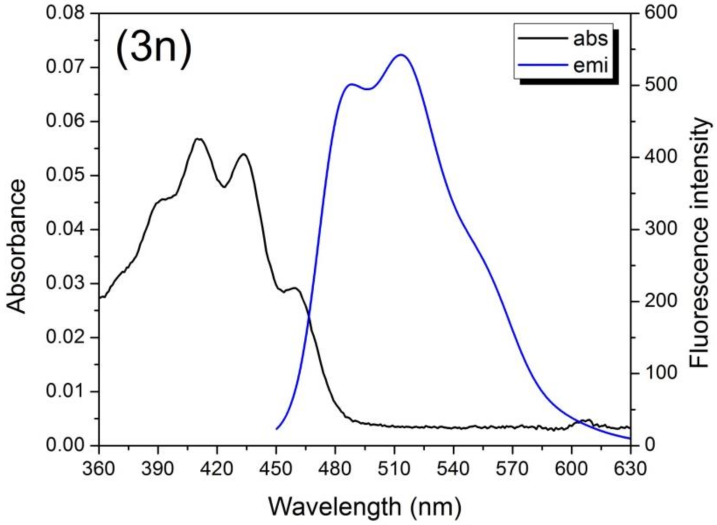
(3n)
abs
emi
Absorbance
Fluorescence intensity
Wavelength (nm)
0.00
0.01
0.02
0.03
0.04
0.05
0.06
0.07
0.08
0
100
200
300
400
500
600
360
390
420
450
480
510
540
570
600
630

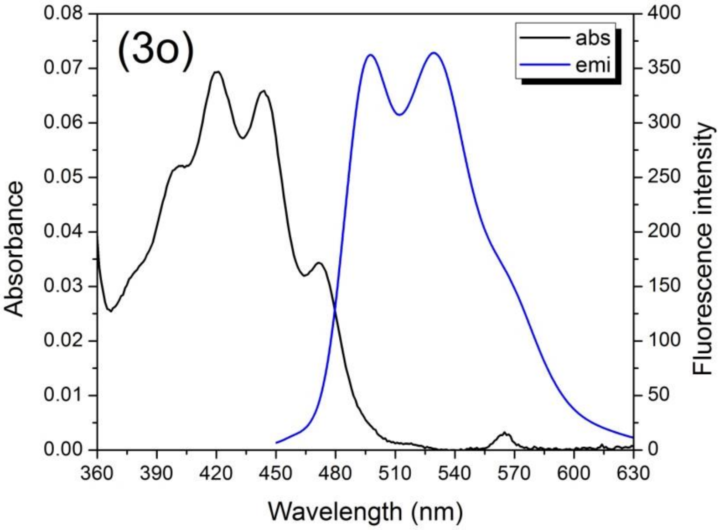
(3o)
abs
emi
Absorbance
Fluorescence intensity
Wavelength (nm)
0.00
0.01
0.02
0.03
0.04
0.05
0.06
0.07
0.08
0
50
100
150
200
250
300
350
400
360
390
420
450
480
510
540
570
600
630

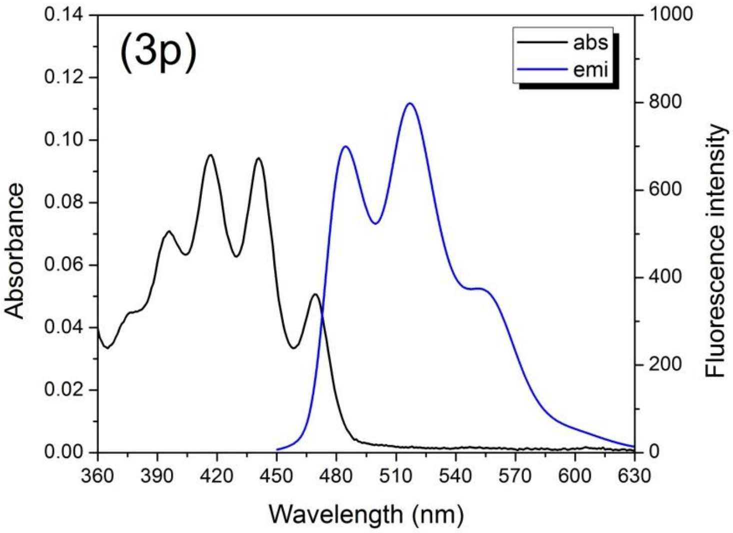
(3p)
abs
emi
Absorbance
Fluorescence intensity
Wavelength (nm)
0.00
0.02
0.04
0.06
0.08
0.10
0.12
0.14
0
200
400
600
800
1000
360
390
420
450
480
510
540
570
600
630

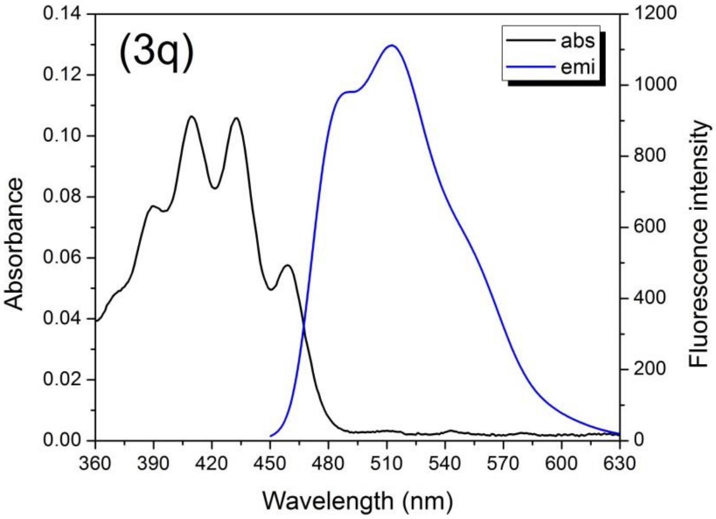
(3q)
abs
emi
Absorbance
Fluorescence intensity
Wavelength (nm)
0.00
0.02
0.04
0.06
0.08
0.10
0.12
0.14
0
200
400
600
800
1000
1200
360
390
420
450
480
510
540
570
600
630

Absorption and fluorescence spectra for studied compounds in various solvents

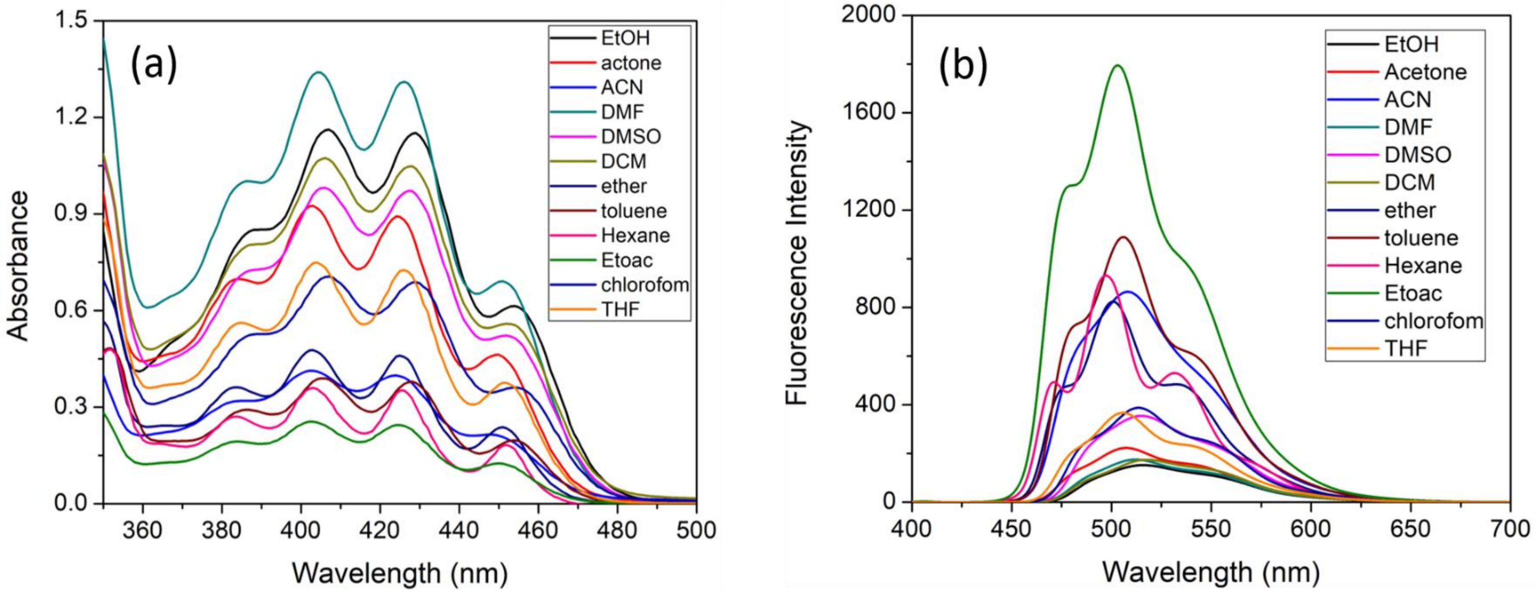

Figures show (a) absorption and (b) emission spectrum of compound **3a** in different solvents
